# Comparison of drug‐coated balloon angioplasty versus common balloon angioplasty for arteriovenous fistula stenosis: A systematic review and meta‐analysis

**DOI:** 10.1002/clc.24078

**Published:** 2023-07-07

**Authors:** Yong Zhang, Fan‐Li Yuan, Xiang‐Yang Hu, Qi‐Bing Wang, Zhen‐Wu Zou, Zhen‐Guo Li

**Affiliations:** ^1^ Department of Nephrology Jianli People's Hospital Jingzhou China; ^2^ Department of Nephrology The First Affiliated Hospital of Yangtze University Jingzhou China; ^3^ Department of Emergency The Central Hospital of Enshi Tujia and Miao Autonomous Prefecture Enshi China; ^4^ Department of General Practice The Central Hospital of Enshi Tujia and Miao Autonomous Prefecture Enshi China; ^5^ Department of Pediatrics Jianli People's Hospital Jingzhou China

**Keywords:** all‐cause mortality, arteriovenous fistula, common balloon angioplasty, drug‐coated balloon, end‐stage renal disease, meta‐analysis, stenosis

## Abstract

Drug‐coated balloons (DCBs) have been used in dialysis patients with arteriovenous fistula (AVF) stenosis, but whether DCBs have advantages over ordinary balloons is still controversial. A meta‐analysis was designed to investigate the safety and efficacy of DCBs and common balloons (CBs) in the treatment of AVF stenosis. We searched the PubMed, EMBASE, and China National Knowledge Internet (CNKI) databases for randomized controlled trials that evaluated the comparison of DCB angioplasty versus CB angioplasty for AVF stenosis in dialysis patients and reported at least one outcome of interest. The results showed that the DCB group had a higher first‐stage patency rate of the target lesion 6 months [odds ratio, OR = 2.31, 95% confidence interval, CI: (1.69, 3.15), *p* < .01] and 12 months [OR = 2.09, 95% CI: (1.50, 2.91), *p* < .01] after surgery. There was no statistically significant difference in all‐cause mortality between the two groups at 6 months [OR = 0.85, 95% CI: (0.47, 1.52), *p* = .58] and 12 months [OR = 0.99, 95% CI: (0.60, 1.64), *p* = .97]. Compared with CB, DCBs as a new endovascular treatment for AVF stenosis have a higher primary patency rate of target lesions and can delay the occurrence of restenosis. There is no evidence that DCB can increase the mortality of patients.

## Graphical abstract

Drug‐coated balloons containing paclitaxel can improve the patency rate of treatment by inhibiting intimal hyperplasia. When paclitaxel‐coated balloons are used to dilate stenotic blood vessels, the balloon can rapidly release drugs that enter the vessel wall quickly, inhibit the proliferation of smooth muscle, and prevent restenosis.

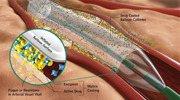

## 1 INTRODUCTION

Worldwide, approximately 2 million people undergo dialysis for end‐stage renal disease,^1^ which is usually treated with hemodialysis.^2^ Autogenous arteriovenous fistula (AVF) is the preferred vascular access for hemodialysis in patients^3^; however, with gradual increases in the application time of AVF, the incidence of vascular access‐related complications such as AVF stenosis or even occlusion increases significantly. Several studies have shown that the AVF patency rate is usually only 60%–65% at 1 year after operation.^4^ An effective solution for AVF stenosis is to perform a second operation to re‐establish the internal fistula, but this will reduce and eventually deplete the vascular resources of dialysis patients. With the development of medicine, percutaneous transluminal angioplasty (PTA) has gradually become one of the main methods for the treatment of AVF stenosis.^5^ However, some studies have suggested that PTA is related to a high restenosis rate of AVF, and intimal hyperplasia is the main pathophysiological mechanism of vascular restenosis after PTA.^6^ A better solution for the treatment of AVF was needed, and a drug‐coated balloon (DCB) approach was developed.

DCBs were initially used for interventional therapy in coronary and lower extremity arteries. The balloon surface is coated with antiproliferative drugs, and the most commonly used drug is paclitaxel. The lipophilicity of paclitaxel increases the drug absorption rate of tissue so that it can effectively inhibit cell migration and proliferation at low concentrations.^7^ When paclitaxel‐coated balloons are used to dilate stenotic blood vessels, the balloon can rapidly release drugs that enter the vessel wall quickly, inhibit the proliferation of smooth muscle, and prevent restenosis.^8^

In recent years, there have been a few studies on the use of DCBs in the treatment of AVF stenosis. However, the sample size of these studies was small, which may lead to bias in interpreting the results.^9^, ^10^ In addition, some randomized controlled trial (RCT) findings are controversial. Common balloon (CB) dilatation is complicated by recurrent restenosis. DCBs containing paclitaxel can improve the patency rate of treatment by inhibiting intimal hyperplasia. This method has been proven to be effective in the coronary artery and femoropopliteal artery, but there are still few studies on vascular access in dialysis. Therefore, we collected studies comparing the safety and efficacy of DCBs and CBs in the treatment of AVF stenosis and conducted a meta‐analysis to provide a reference for clinical decision‐making.

## 2 METHODS

### 2.1 Study protocol

This article was conducted in accordance with the Preferred Reporting Items for Systematic Reviews and Meta‐Analyses (PRISMA) guidelines^11^ and registered in INPLASY (DOI:10.37766/inplasy2022.8.0112).

### 2.2 Search strategy

Two researchers (Yong Zhang and Xiang‐Yang Hu) independently searched the PubMed, EMBASE, and China National Knowledge Internet (CNKI) databases from inception to August 11, 2022, by using Medical Subject Headings (MeSH), Emtree, and text words with no language limitations.

The following keywords were used for the search strategy: “Drug‐coated balloon,” “DCB,” “Paclitaxel‐coated balloon,” “Haemodialysis access,” “Vascular access,” “Arteriovenous fistula,” “AVF,” “dialysis,” “haemodialysis”, “Angioplasty,” and “stenosis.” Reference lists from the identified studies were also searched for potentially eligible articles. Preliminary publications were imported into EndNote X9.1 (Clarivate Analytics), duplicate records and irrelevant studies were removed, and appropriate studies with detailed classification were compiled (Supporting Information: Table S2).

### 2.3 Eligibility criteria

Two authors (Yong Zhang and Xiang‐Yang Hu) independently carried out the primary review to search for trials that met the inclusion criteria (Supporting Information: Table S3). Any discrepancy was resolved by discussion and consensus (Supporting Information: Figure S1). The following criteria were used: (1) Adult participants (≥18 years) on dialysis for at least 6 months, irrespective of age, sex, and race; (2) comparison of hemodialysis patients with stenotic AVF or arteriovenous graft (AVG) who underwent DCB angioplasty and those who underwent CB angioplasty at 6 months or 12 months; (3) inclusion of one of the following outcomes: primary patency and all‐cause mortality at 6 or 12 months; (4) only RCTs were included in the meta‐analysis. The main characteristics of the included studies are listed in Table 1 and Supporting Information: Table S1.

**Table 1:** Main characteristics of the included studies.

References	Country	Sample		DCB versus CB^a^		Average age		Dose^b^	Follow‐up	Result
		DCB	CB	6 months	12 months	DCB	CB	(ug/mm^2^)	(months)	
Bjorkman et al.^13^	Finland	18	18	N	16/4	67	67.4	3.5	12	①②④
Fransson et al.^18^	Sweden	22	20	10/8	4/2	68	62	N	12	②③④
Guo et al.^14^	China	20	22	17/12	14/10	63.3 ± 10.7	59.9 ± 11.7	N	12	①②
Irani et al.^10^	Singapore	59	60	48/37	30/20	59.0 ± 11.5	59.4 ± 8.80	3.0	12	③④
Kitrou et al.^19^	United Kingdom	20	20	15/0	10/0	64.3 ± 14.5	57 ± 14.2	3.0	12	③④
Kitrou et al.^9^	United Kingdom	20	20	8/4	2/1	56.7	57	2	6	①②③④
Lai et al.^20^	China Taiwan	10	10	7/0	2/0	67.2 ± 9.4		N	12	③④
Liao et al.^21^	China Taiwan	22	22	9/2	8/2	70.4 ± 10.6	65.9 ± 15.9	N	12	③④
Lookstein et al.^22^	New Zealand	170	160	125/88	N	65.8 ± 13.1	65.5 ± 13.4	3.5	6	①③
Maleux et al.^23^	Belgium	33	31	22/20	14/12	66.9 ± 17.0	69.3 ± 14.85	N	12	①②③④
Moreno et al.^24^	Spain	71	69	57/45	41/37	71 ± 11.31	69 ± 12.99	N	12	②③④
Roosen et al.^25^	Netherlands	16	18	2/8	1/2	80	83	N	24	③④
Swinne et al.^15^	Australia	68	60	52/28	25/14	65.2 ± 13.6	64.5 ± 13.9	3	12	③④
Trerotola et al.^18^	America	141	144	100/91	N	64 ± 15	61 ± 13	2	6	③④
Yin et al.^17^	China	78	83	51/31	57/47	56 ± 13	54 ± 13	3	12	②③

*Note*: ①, all‐cause mortality at 6 months; ②, all‐cause mortality at 12 months; ③, primary patency rate of the target lesion at 6 months after operation; ④, primary patency rate of the target lesion 21 months after the operation.

Abbreviations: CB, common balloon; DCB, drug‐coated balloon; N, not clear.

^a^ Target lesion primary patency.

^b^ Paclitaxel dose.

### 2.4 Data extraction

Two reviewers (Xiang‐Yang Hu and Yong Zhang) independently extracted data from the same set of publications. The following information was extracted: author, year, country, sample size, average age, paclitaxel dose, follow‐up, and main results.

### 2.5 Summary of effect size

Odds ratios (ORs) with 95% confidence intervals (CIs) were used as the effect size measures of dichotomous data. The weight of enrolled studies was determined by accounting for the size of the treatment group, control group, and total sample size. A *Z*‐test was calculated, and therapeutic efficacy was deemed significant with a *p* < .05 cut‐off.^12^

### 2.6 Risk of bias

The quality of all trials was independently evaluated by two authors (Xiang‐Yang Hu and Yong Zhang) according to the Cochrane quality criteria (Supporting Information: Figure S2). Any disagreement between the authors was settled by a discussion with a third author (Zhen‐Wu Zou). A weighted kappa value was calculated to examine agreement between reviewers for the overall study risk of bias assessment (Supporting Information: Table S5).

### 2.7 GRADE quality assessment

The overall quality of evidence was evaluated by two authors (Fan‐Li Yuan and Yong Zhang) according to The Grading of Recommendations Assessment, Development, and Evaluation (GRADE) criteria. The results and the overall quality of evidence are presented in Supporting Information: Table S4.

### 2.8 Statistical analysis

STATA 16.0 (Stata Corp LP) was used to perform statistical analyses. L'Abbe plots and meta‐regression were used for intuitive judgment of heterogeneity. For the remaining circumstances, a random effect model was used to pool the effect size to calculate statistical heterogeneity. Heterogeneity was analyzed by *I* ^2^ and *χ* ^2^ statistics. If there was significant heterogeneity, a L'Abbe plot and Galbraith plot were generated to evaluate the consistency and quality of the results. Sensitivity analysis, subgroup analysis, and meta‐regression were performed to determine sources of heterogeneity. Publication bias was evaluated using Begg's and Egger's tests and funnel plots.

## 3 RESULTS

### 3.1 Study selection

A total of 484 studies were identified during the initial search after excluding duplicate records (*n* = 35). Eighty‐five articles were retained after title/abstract curation (excluding 433 records). Thereafter, we read the full text and analyzed 15 RCTs^9^, ^10^, ^13^, ^14^, ^15^, ^16^, ^17^, ^18^, ^19^, ^20^, ^21^, ^22^, ^23^, ^24^, ^25^ involving a total of 1525 patients for quantitative synthesis (Supporting Information: Figure S1). The main characteristics of the included RCTs (country, design, sample size, age, intervention, follow‐up, and main results) are described in Table 1.

### 3.2 Primary patency rate of the target lesion

**Figure 1:**
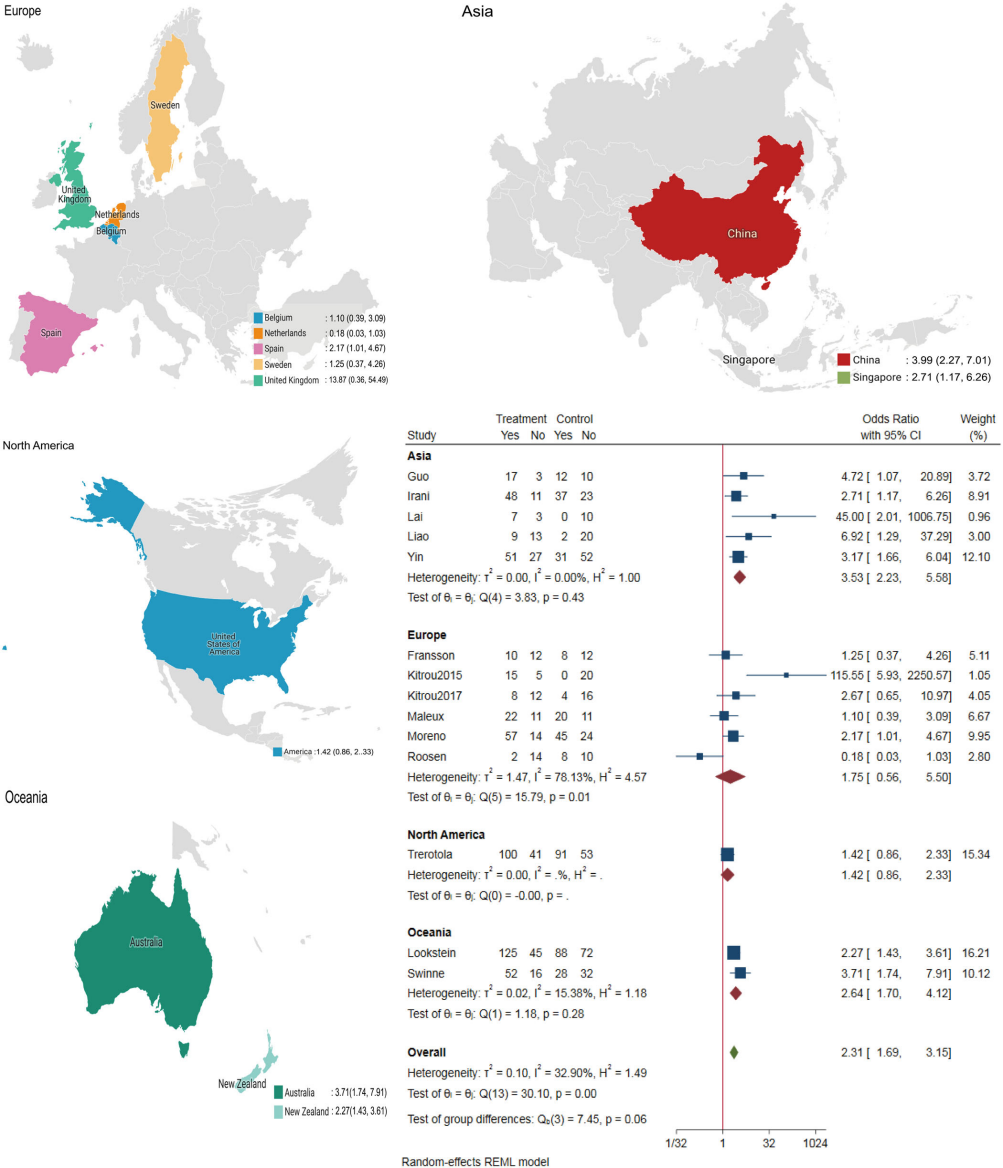
The choropleth map of the 6‐month primary patency rate. CI, confidence interval.

Pooled analysis of 14 studies^9^, ^10^, ^14^, ^15^, ^16^, ^17^, ^18^, ^19^, ^20^, ^21^, ^22^, ^23^, ^24^, ^25^ (*n* = 1489) using a forest plot demonstrated that the primary patency rate in the DCB group (*n* = 750) was higher than that in the CB group (*n* = 739) at 6 months after balloon expansion (OR = 2.31, 95% CI: 1.69, 3.15, *p* < .01, Supporting Information: Figure S3). No significant heterogeneity was observed (*I* ^2^ = 32.90%). The L'Abbe plot showed that 13 of the 14 included studies fell to the upper left of the diagonal line, indicating a higher primary patency rate in the DCB group (Supporting Information: Figure S4). The choropleth map revealed regional differences in the 6‐month primary patency rate between Belgium, the Netherlands, Sweden, the United Kingdom, and the United States of America (Figure 1). Overall, subgroup analysis showed that in Asia and Oceania, DCBs had a higher primary patency rate at 6 months after balloon expansion, but in Europe and North America, there was no significant difference between DCBs and CBs (Supporting Information: Figure S5). The meta‐regression by bubble plot revealed no significant heterogeneity in the study size (*p* = .557, Supporting Information: Figure S6) OR publication year (*p* = .816, Supporting Information: Figure S7). Sensitivity analysis (Supporting Information: Figure S8) was performed, and Galbraith plots (Supporting Information: Figure S9) were generated to evaluate the stability of our results. The analysis suggested that no individual studies significantly affected the pooled OR, indicating that the results were statistically robust. No significant publication bias was found by Begg's plots (*p* = .228, Supporting Information: Figure S10), Egger's test (*p* = .271, Supporting Information: Figure S11) OR funnel plots (Supporting Information: Figure S12).

**Figure 2:**
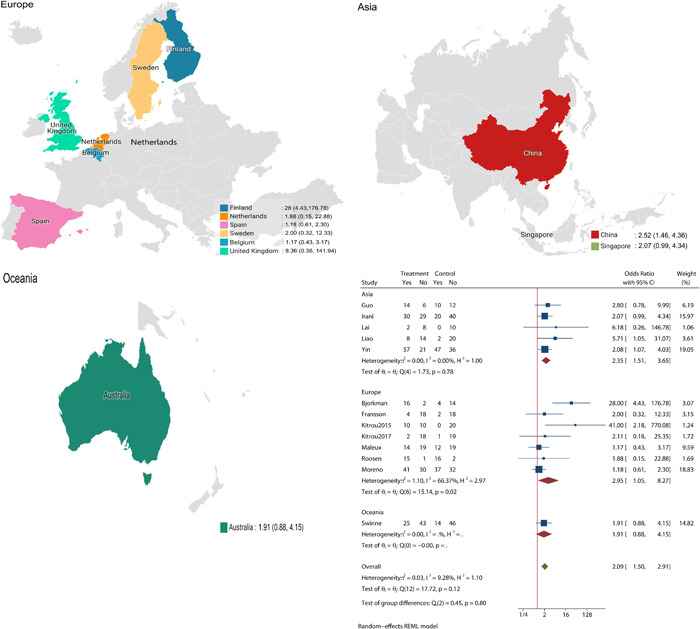
The choropleth map of the 12‐month primary patency rate. CI, confidence interval.

Pooled analysis of 13 studies^9^, ^10^, ^13^, ^14^, ^15^, ^17^, ^18^, ^19^, ^20^, ^21^, ^23^, ^24^, ^25^ (*n* = 910) using a forest plot demonstrated that the primary patency rate at 12 months after balloon expansion in the DCB group (*n* = 457) was higher than that in the CB group (*n* = 453) (OR = 2.09, 95% CI: 1.50, 2.91, *p* < .01, Supporting Information: Figure S13). No significant heterogeneity was observed (*I* ^2^ = 9.28%). The L'Abbe plot showed that all 13 included studies fell to the upper left of the diagonal line, also indicating a higher primary patency rate at 12 months in the DCB group (Supporting Information: Figure S14). The choropleth map revealed regional differences in the 12‐month primary patency rate in Australia, Belgium, the Netherlands, Sweden, Singapore, Spain, and the United Kingdom (Figure 2). Overall, subgroup analysis showed that in Asia and Europe, DCBs had a higher primary patency rate at 12 months after balloon expansion, but in Oceania, there was no significant difference between DCBs and CBs (Supporting Information: Figure S15). The meta‐regression by bubble plot analysis revealed no significant heterogeneity in the study size (*p* = .067, Supporting Information: Figure S16) OR publication year (*p* = .597, Supporting Information: Figure S17). Sensitivity analysis (Supporting Information: Figure S18) was performed, and Galbraith plots (Supporting Information: Figure S19) were generated to evaluate the stability of our results. The analysis suggested that no individual studies significantly affected the pooled OR, indicating that the results were statistically robust. Moreover, no significant publication bias was found by Begg's plots (*p* = .161, Supporting Information: Figure S20), Egger's test (*p* = .942, Supporting Information: Figure S21) OR funnel plots (Supporting Information: Figure S22).

### 3.3 All‐cause mortality

**Figure 3:**
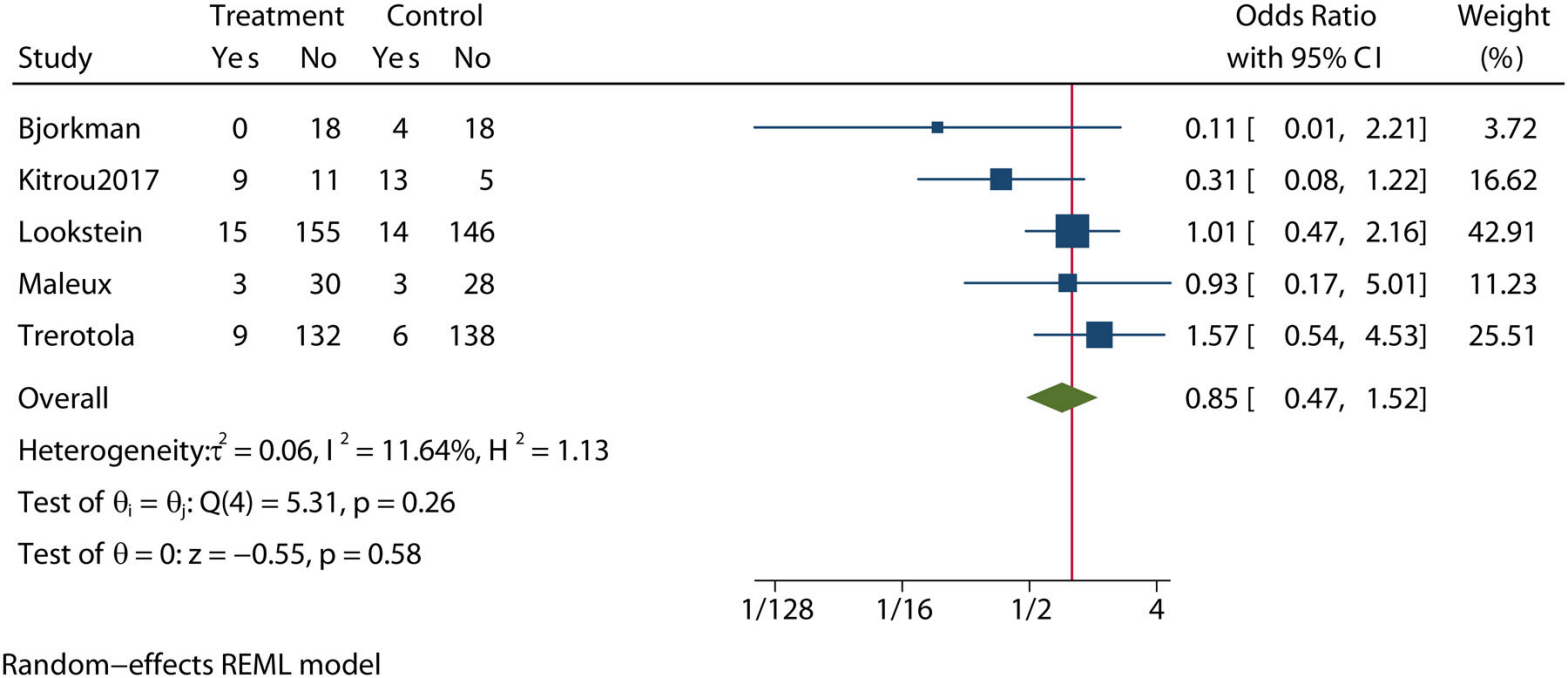
Forest plot of all‐cause mortality at 6 months after balloon expansion. CI, confidence interval.

Five studies^9^, ^13^, ^16^, ^22^, ^23^ assessed the incidence of all‐cause mortality at 6 months after balloon expansion between the two groups in a total of 757 patients, with 382 assigned to the DCB group and 375 assigned to CB group (OR = 0.85, 95% CI: 0.47, 1.52, *p* = .58, Figure 3). No significant heterogeneity was observed (*I* ^2^ = 11.64%). The meta‐regression by bubble plot revealed no significant heterogeneity of the sample size (*p* = .090, Supporting Information: Figure S23) OR publication year (*p* = .476, Supporting Information: Figure S24). Sensitivity analysis (Supporting Information: Figure S25) was performed, and Galbraith plots (Supporting Information: Figure S26) were generated to evaluate the stability of our results and we found that no individual studies significantly affected the pooled OR, confirming that the results were statistically robust. No significant publication bias was found by Begg's plots (*p* = .327, Supporting Information: Figure S27), Egger's test (*p* = .318, Supporting Information: Figure S28) OR funnel plot (Supporting Information: Figure S29).

**Figure 4:**
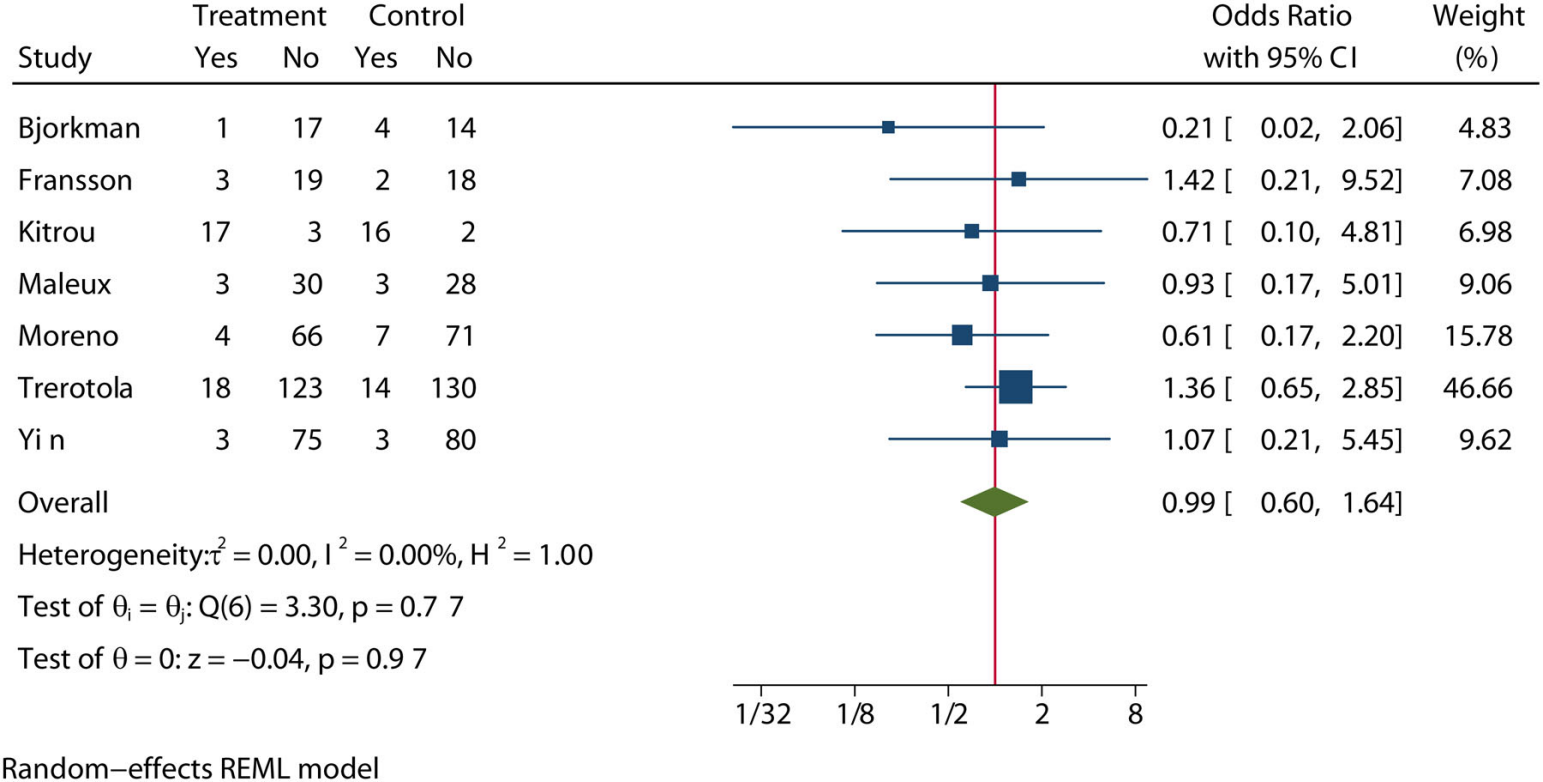
Forest plot of all‐cause mortality of 12 months after balloon expansion.

Seven studies^9^, ^13^, ^16^, ^17^, ^18^, ^23^, ^24^ assessed the incidence of all‐cause mortality at 12 months after balloon expansion between the two groups in a total of 774 patients, with 382 assigned to DCB groups and 392 assigned to CB groups (OR = 0.99, 95% CI: 0.60, 1.64, *p* = .97, Figure 4). No significant heterogeneity was observed (*I* ^2^ = 0%). The meta‐regression by bubble plot revealed no significant heterogeneity of the sample size (*p* = .241, Supporting Information: Figure S30) OR publication year (*p* = .912, Supporting Information: Figure S31). Sensitivity analysis (Supporting Information: Figure S32) and Galbraith plots (Supporting Information: Figure S33) were performed to evaluate the stability of our results. The analysis results suggested that no individual studies significantly affected the pooled OR, indicating that the results were statistically robust. No significant publication bias was found in the results of Begg's plots (*p* = .453, Supporting Information: Figure S34), Egger's test (*p* = .793, Supporting Information: Figure S35) OR the funnel plot (Supporting Information: Figure S36).

## 4 DISCUSSION

### 4.1 Main findings

Contrary to previous studies,^26^ our meta‐analysis demonstrated that DCBs had a significantly higher primary patency rate of target lesions than CBs at 6 and 12 months after angioplasty. Meanwhile, we observed that there were some regional differences in the above conclusions. Overall, DCB has a higher primary patency rate at 6 months after balloon expansion in Asia and Oceania, but in Europe and North America, there is no significant difference between DCB and CB treatment. DCB has a higher primary patency rate at 12 months after balloon expansion in Asia and Europe, but in Oceania, there is no significant difference between the two groups. There could be many reasons for these regional differences, such as degree of stenosis, primary stenosis, central vein stenosis, diabetes mellitus, hypertension, antiplatelet aggregation drug use, drug dosage, and so on. We did not perform subgroup analyses sequentially, which may have affected our results. There was no statistically significant difference in all‐cause mortality between the two groups at 6 and 12 months.

### 4.2 Interpretation

Most patients with end‐stage renal disease require long‐term hemodialysis treatment, and vascular access is essential for effective hemodialysis. At present, long‐term hemodialysis treatment mainly relies on AVF and AVG. Among them, AVF has become the first choice for dialysis access due to its advantages of long application time and fewer intervention times.^27^ However, with the gradual prolongation of the application time of AVF, the incidence of vascular access‐related complications such as AVF stenosis or even occlusion has increased significantly. Several studies have shown that the AVF patency rate after 1 year is usually only 60%–65%.^4^ A large number of studies have shown that the main mechanism of AVF stenosis is that under the pro‐inflammatory state of chronic kidney disease, hemodynamic changes caused by different anatomical morphologies of dialysis access and repeated puncture promote the proliferation of vascular intima tissue, make the vascular lumen smaller, and eventually lead to the failure of dialysis access.^28^ An effective solution to AVF stenosis is to use secondary surgery to re‐establish the internal fistula, but this will reduce the vascular resources of dialysis patients and eventually lead to the depletion of vascular resources.

With the advancement of medicine, PTA has gradually become one of the main methods for the treatment of AVF stenosis. In recent years, high‐pressure balloons, cutting balloons, and other instruments have been gradually used in clinical practice, but there is still a high incidence of restenosis, as high as 55%–75% within 1 year.^24^ It has been suggested that PTA is associated with a high restenosis rate of AVF, and intimal hyperplasia is the main pathophysiological mechanism of restenosis after PTA.^6^ A better solution was needed, so a DCB approach was developed.

DCB was originally used in coronary and lower extremity arterial interventional therapy, with drugs that inhibit cell proliferation attached to the surface of balloons. At present, paclitaxel is the most commonly used drug for DCB. The lipophilicity of paclitaxel can increase the tissue drug absorption rate so that it can effectively inhibit cell migration and proliferation at low concentrations.^7^ When paclitaxel‐coated balloons are used to dilate stenotic blood vessels, the balloon can rapidly release drugs, make drugs enter the vessel wall quickly, inhibit the proliferation of smooth muscle, and prevent restenosis.^8^

In recent years, there have been a few studies on the use of DCBs in the treatment of AVF stenosis. However, the sample size of these studies was small, which may lead to bias in the results.^10^ In addition, some RCT findings are also controversial. This meta‐analysis showed that compared with CB angioplasty, DCB angioplasty had a higher primary patency rate of target lesions at 6 and 12 months after the operation, and the difference was statistically significant (*p* < .05). This confirmed that DCB could delay the occurrence of restenosis of AVF, improve the primary patency rate of target lesions, and thus prolong the vascular access time of patients with end‐stage renal disease.

In addition, studies have shown that the use of paclitaxel DCB can increase the risk of death in patients with peripheral artery disease, which has aroused widespread concern about the safety of paclitaxel‐related endovascular devices.^29^ However, some scholars questioned whether the above analysis results were affected by selection bias.^30^ Recently, a large number of studies have shown that paclitaxel DCB is safe, and there is no significant correlation between the use of paclitaxel and the death of patients.^31^, ^32^ In terms of the safety of paclitaxel DCB in AVF stenosis, the results of this study showed that there was no significant difference in all‐cause mortality between the DCB group and the POBA group at 6 and 12 months after the operation (*p* > .05). Therefore, we believe that there is currently no evidence that paclitaxel DCB increases mortality in patients with AVF.

However, it is worth noting that although DCBs can prolong the primary patency rate of target lesion pairs without increasing the mortality rate of patients, the wide application of DCBs in the treatment of AVF stenosis is limited due to the large difference in price between DCBs and CB, their high initial treatment cost, and unclear potential economic benefits.

## 5 STRENGTHS AND LIMITATIONS

First, our meta‐analysis was performed by strict quality control evaluated by Cohen's *κ* coefficient (*κ* = 0.823, 95% CI: 0.642–0.937), which showed a high degree of agreement. Second, contrary to previous studies,^26^ our study included more higher quality RCTs (*n* = 15), and we attempted to be as inclusive and transparent in this manuscript in terms of our methods, including all origins of software and websites. Third, we refined our analyses strictly in accordance with the PICOS principle. We performed rigorous statistical analysis. For instance, the L'Abbe plot and Galbraith plot were used to evaluate the consistency and quality of the results. Sensitivity analysis, meta‐regression, and subgroup analysis with a choropleth map were performed to determine sources of heterogeneity. Publication bias was evaluated using Begg's and Egger's tests and a funnel plot. Specifically, we conducted four subgroup analyses according to different countries and continents to make our findings more comprehensive and rigorous.

However, several limitations of our meta‐analysis should be considered. First, the true event rates of participants lost to follow‐up are unpredictable and unlikely to be at either extreme of our assumptions. Second, the paclitaxel doses were variable in studies analyzed, which could have affected our conclusions. Third, the concomitant diseases of patients included in various institutes were different, which may lead to a risk of bias. Fourth, this meta‐analysis did not include a particularly large number of RCTs and could have affected our conclusions.

## 6 CONCLUSIONS

In conclusion, compared with CB, DCB as a new endovascular treatment for AVF stenosis has a higher primary patency rate of target lesions and can delay the occurrence of restenosis. At present, there is no evidence that DCB can increase the mortality of patients, but further clinical studies are needed to determine its mid‐ and long‐term efficacy and safety in the treatment of AVF stenosis. We believe that future studies should optimize the application technology according to specific vascular access types or lesion characteristics and clarify the role of DCBs in hemodialysis vascular access management.

## AUTHOR CONTRIBUTIONS

*Conceptualization*: Fan‐Li Yuan, Xiang‐Yang Hu, Qi‐Bing Wang, and Zhen‐Guo Li. *Data curation*: Yong Zhang, Fan‐Li Yuan, and Zhen‐Guo Li. *Formal analysis*: Yong Zhang, Fan‐Li Yuan, and Zhen‐Guo Li. *Investigation*: Fan‐Li Yuan, Zhen‐Wu Zou, and Zhen‐Guo Li. *Methodology*: Yong Zhang, Fan‐Li Yuan, and Zhen‐Guo Li. *Project administration*: Yong Zhang, Fan‐Li Yuan, and Zhen‐Guo Li. *Resources*: Zhen‐Wu Zou and Yong Zhang. *Software*: Yong Zhang, Fan‐Li Yuan, and Zhen‐Guo Li. *Supervision*: Yong Zhang, Fan‐Li Yuan, and Zhen‐Guo Li. *Validation*: Yong Zhang, Fan‐Li Yuan, and Zhen‐Guo Li. *Visualization*: Fan‐Li Yuan, Zhen‐Wu Zou, and Yong Zhang. *Roles/Writing—original draft*: Yong Zhang, Fan‐Li Yuan, and Zhen‐Guo Li. *Writing—review and editing*: Fan‐Li Yuan, Zhen‐Wu Zou, and Yong Zhang.

## CONFLICT OF INTEREST STATEMENT

The authors declare no conflict of interest.

## Supporting information

Supporting information.

Supporting information.

## Data Availability

Data are available upon reasonable request from the authors. The original contributions presented in the study are included in the article/Supporting Information, further inquiries can be directed to the corresponding authors.
