# Supplementary material for: Comparison of drug‐coated balloon angioplasty versus common balloon angioplasty for arteriovenous fistula stenosis: A systematic review and meta‐analysis
Source: Clin Cardiol. 2023 Jul 7;46(8):877–85. doi: 10.1002/clc.24078 (PMC10436783; doi:10.1002/clc.24078)

**Figure legends**

**Fig 1. The choropleth map of the 6-month primary patency rate.**

**
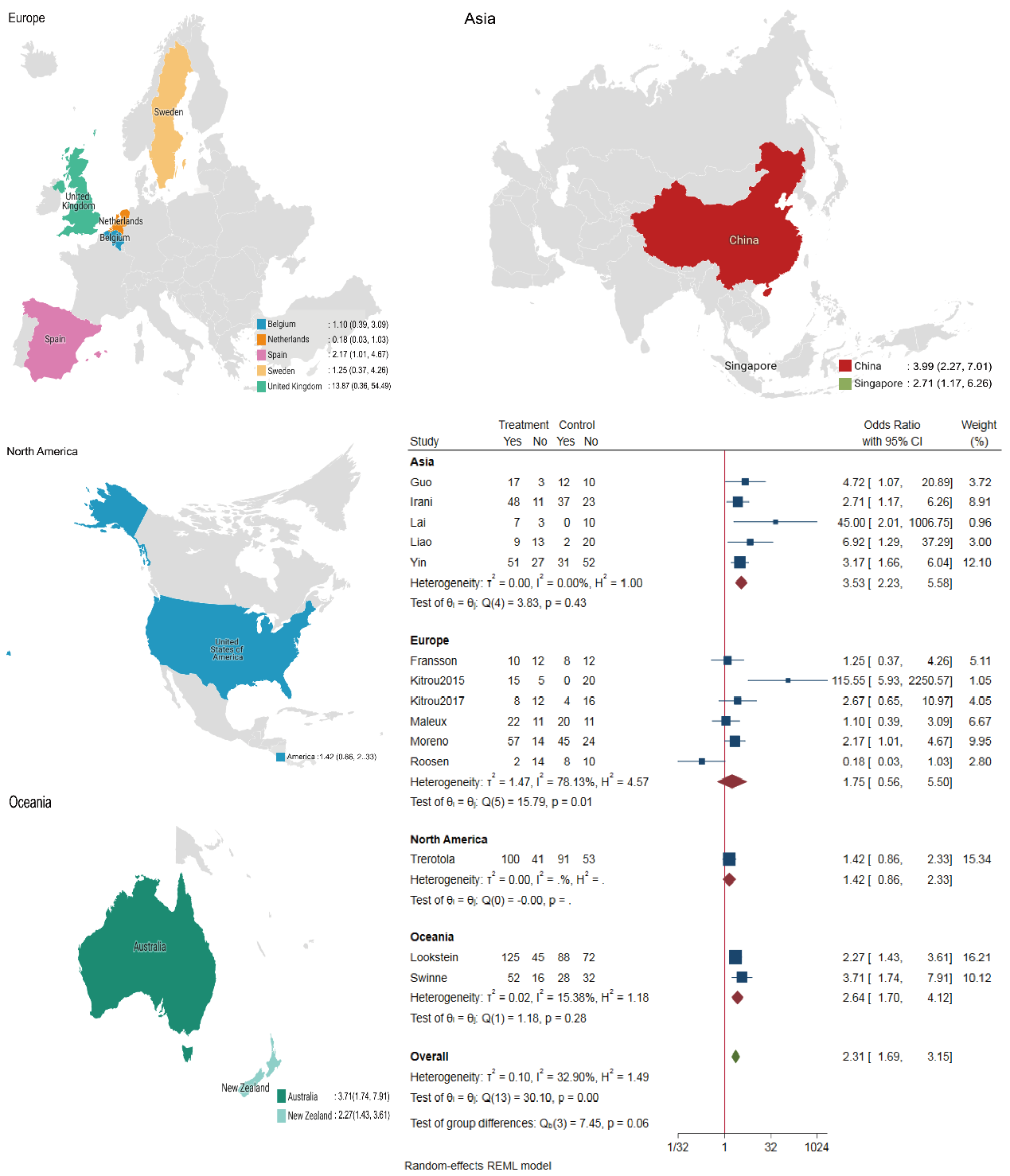
**

**Fig 2. The choropleth map of the 12-month primary patency rate.**

**
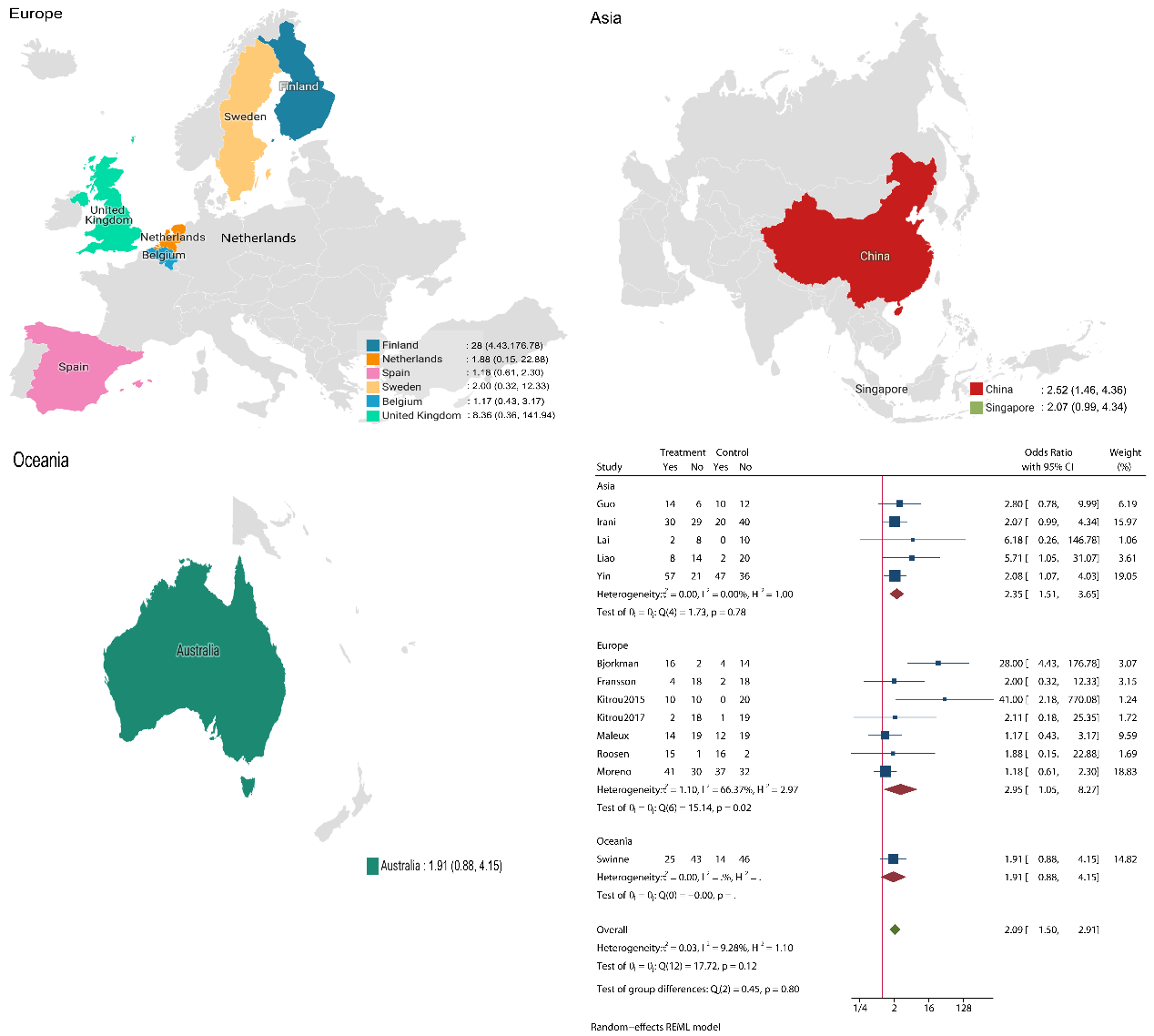
**

**Fig 3. Forest plot of all-cause mortality at 6 months after balloon expansion.**

**
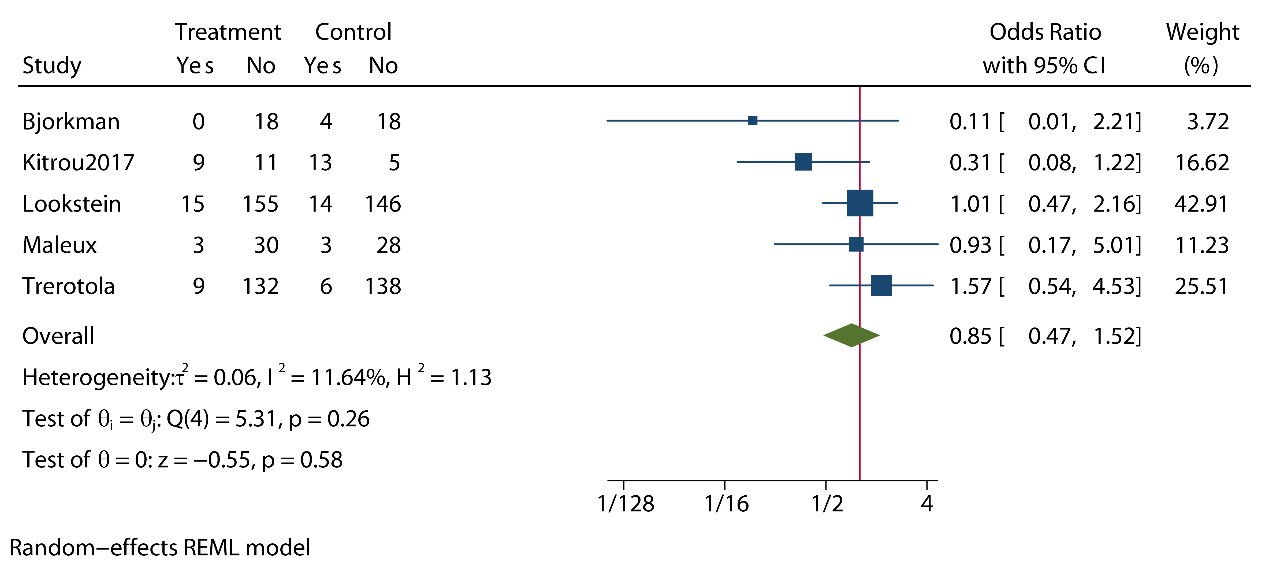
**

**Fig 4. Forest plot of all-cause mortality of 12-months after balloon expansion**

**
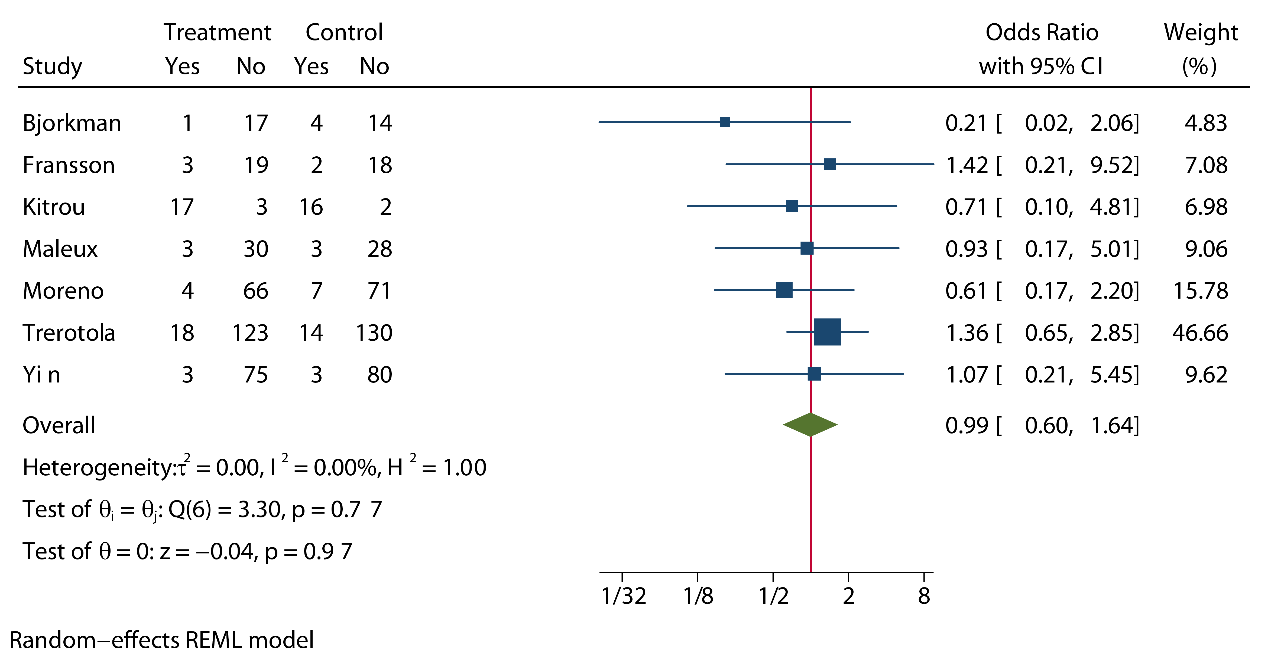
**

**Fig S1. PRISMA 2009 flow diagram.**

**
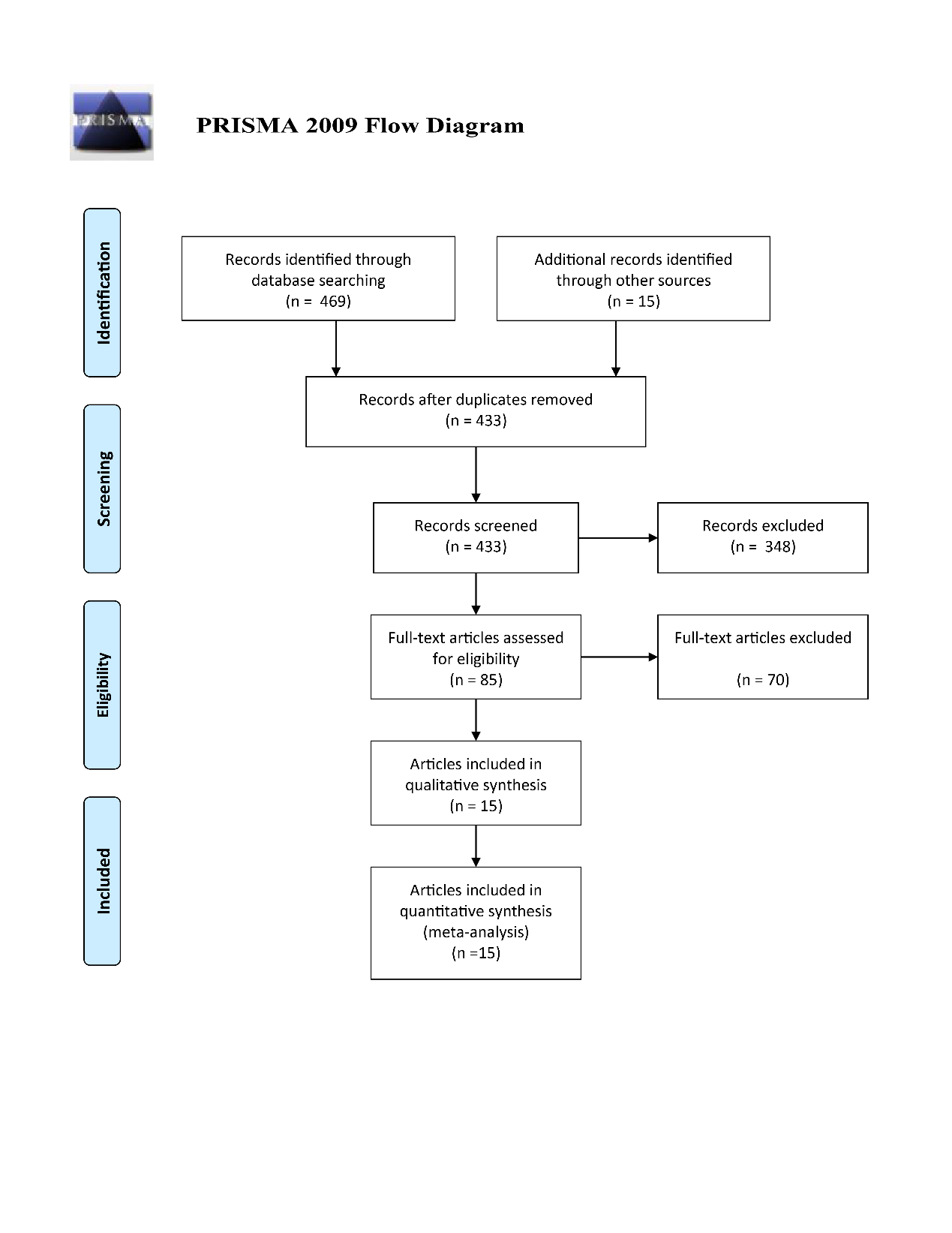
**

**Fig S2. Risk of bias assessments for included studies.**

**
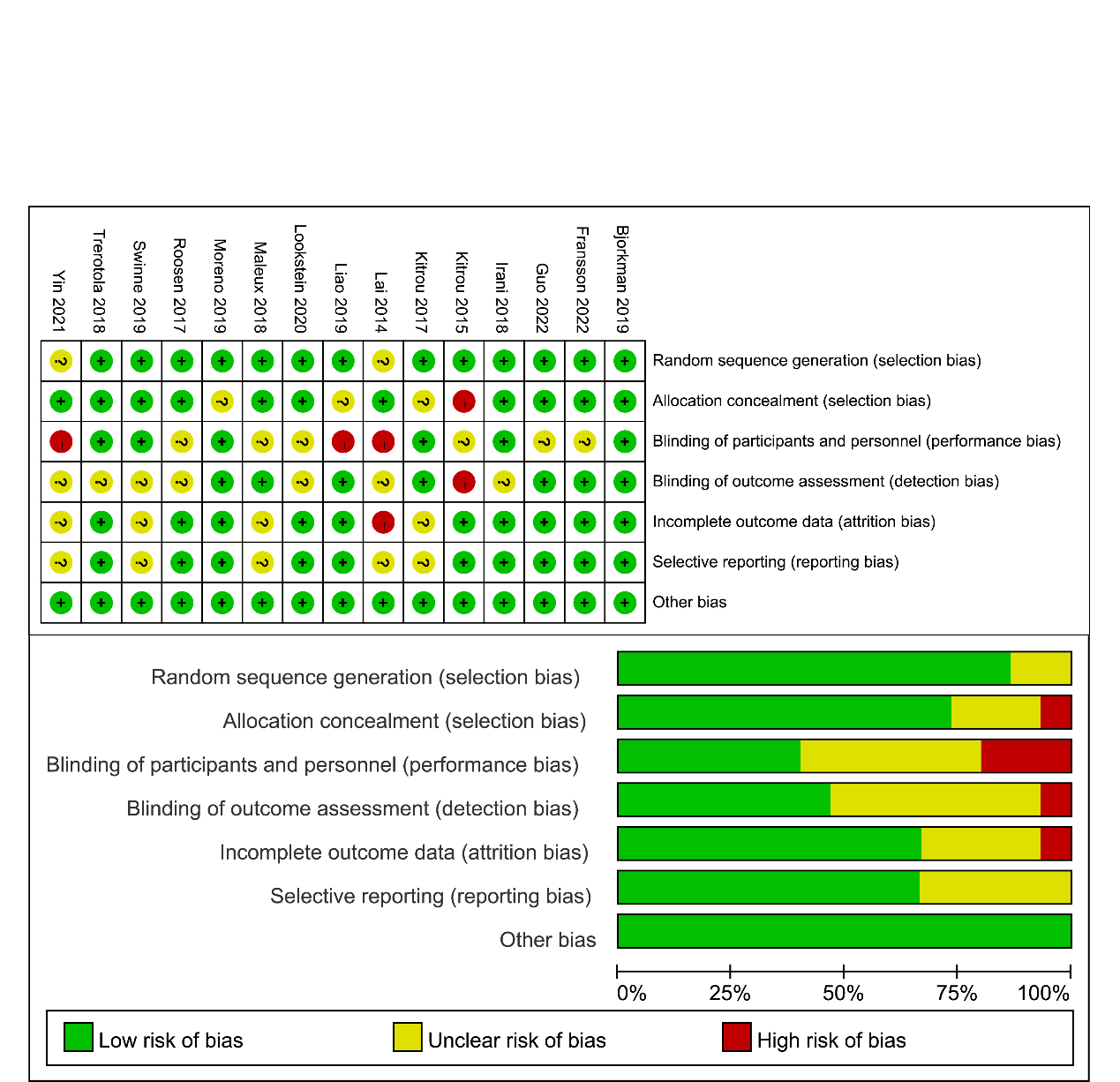
**

**Fig S3. Forest plot of 6-month primary patency of target lesion.**

**
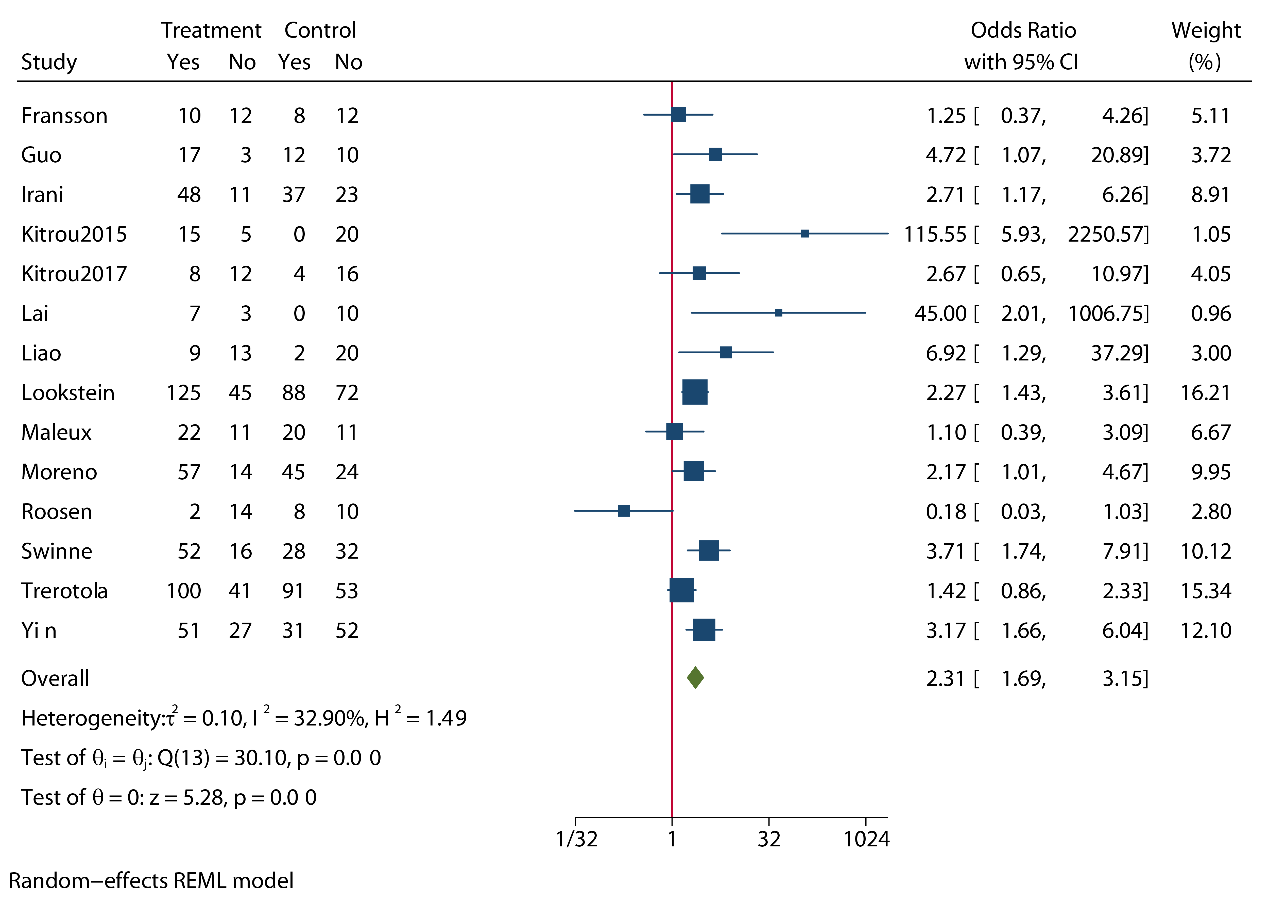
**

**Fig S4. L'Abbe plot of the 6-month primary patency rate.**

**
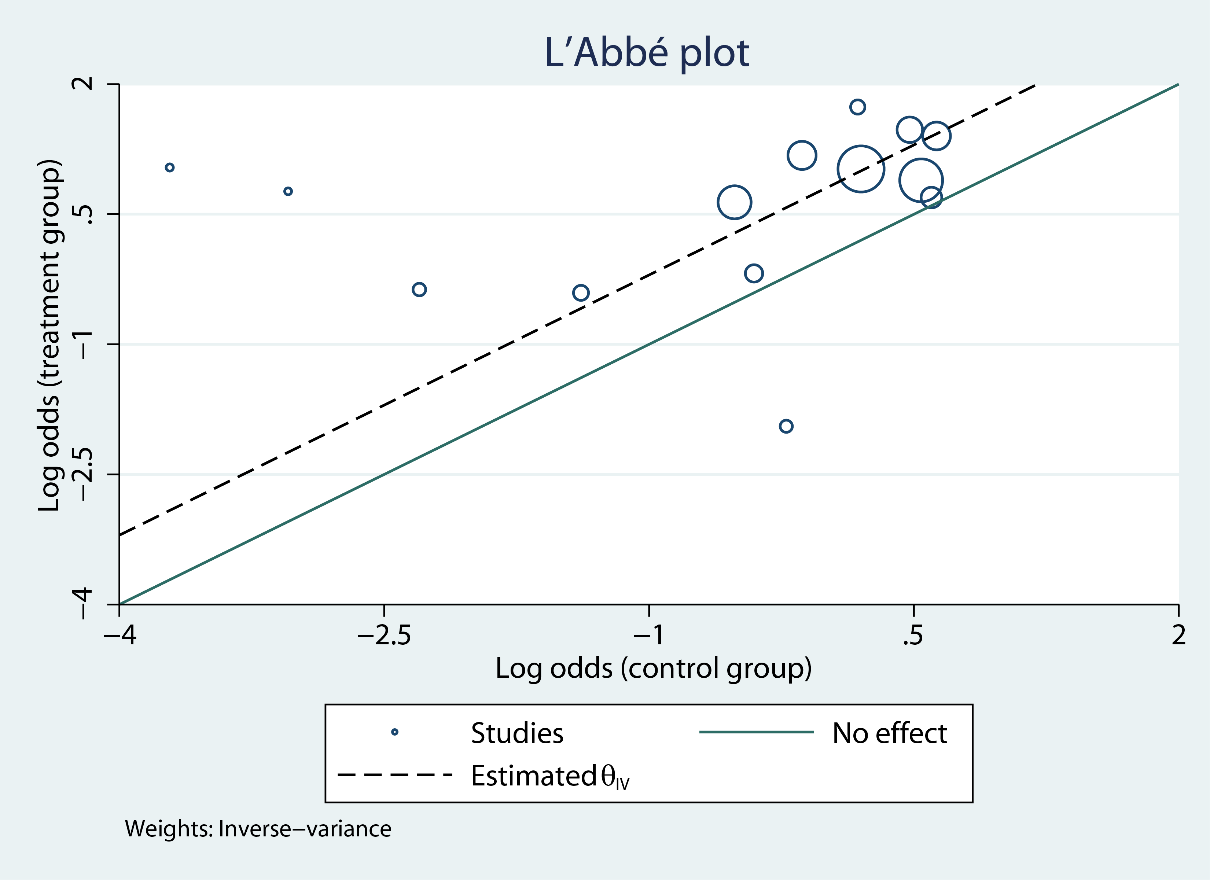
**

**Fig S5. Subgroup analysis of the 6-month primary patency rate.**

**
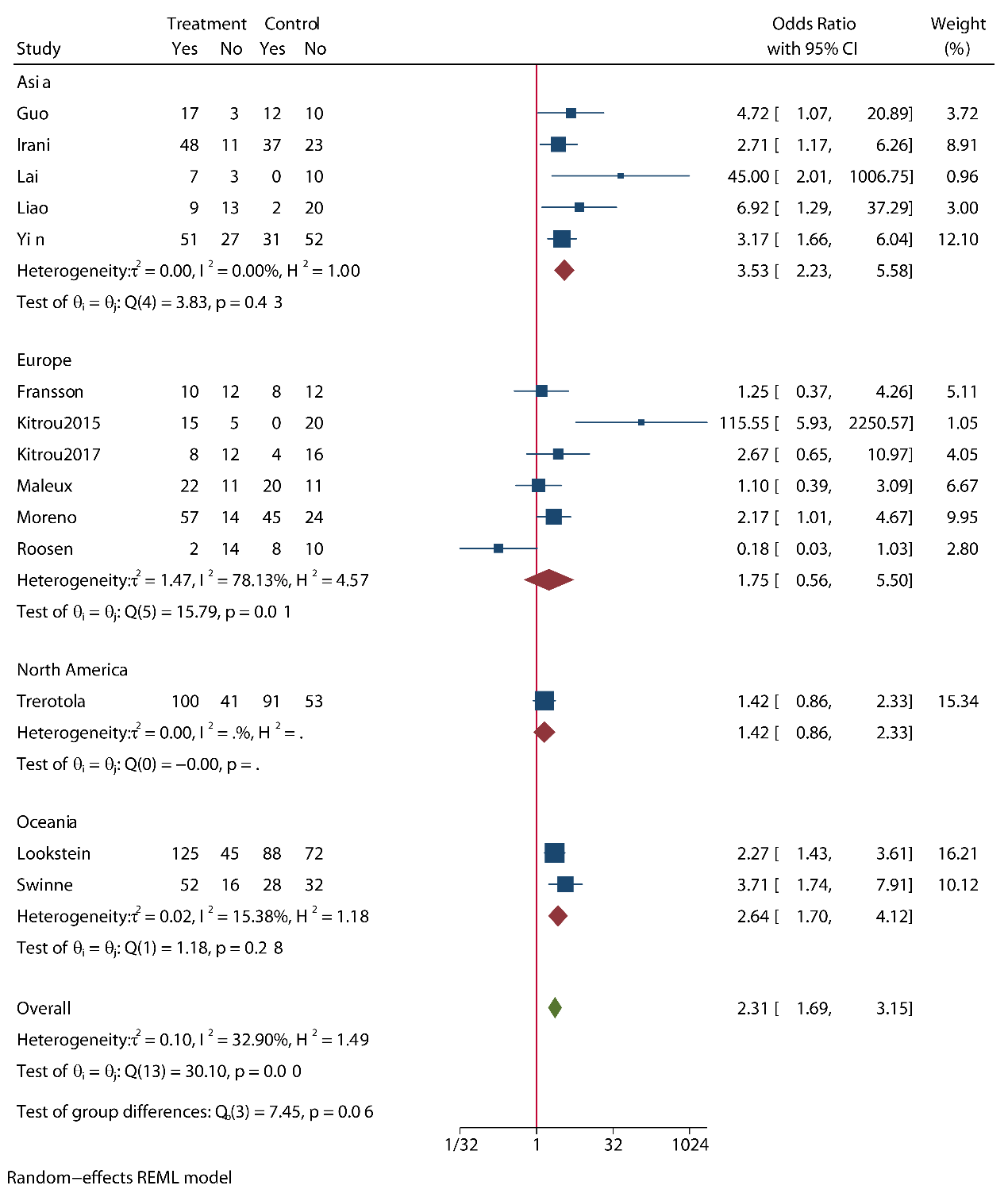
**

**Fig S6. Meta-regression of sample size of 6-month primary patency rate.**

**
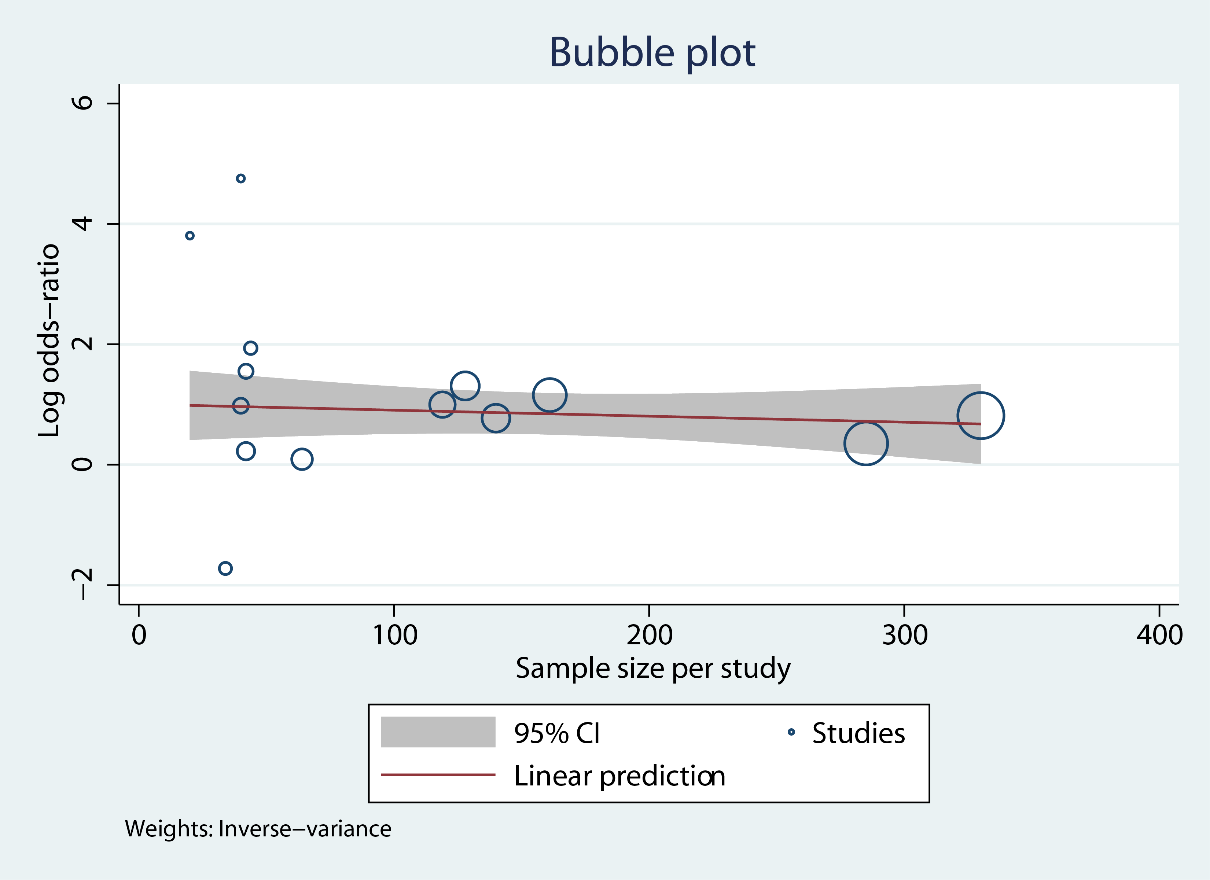
**

**Fig S7. Meta-regression of publication year of 6-month primary patency rate.**

**
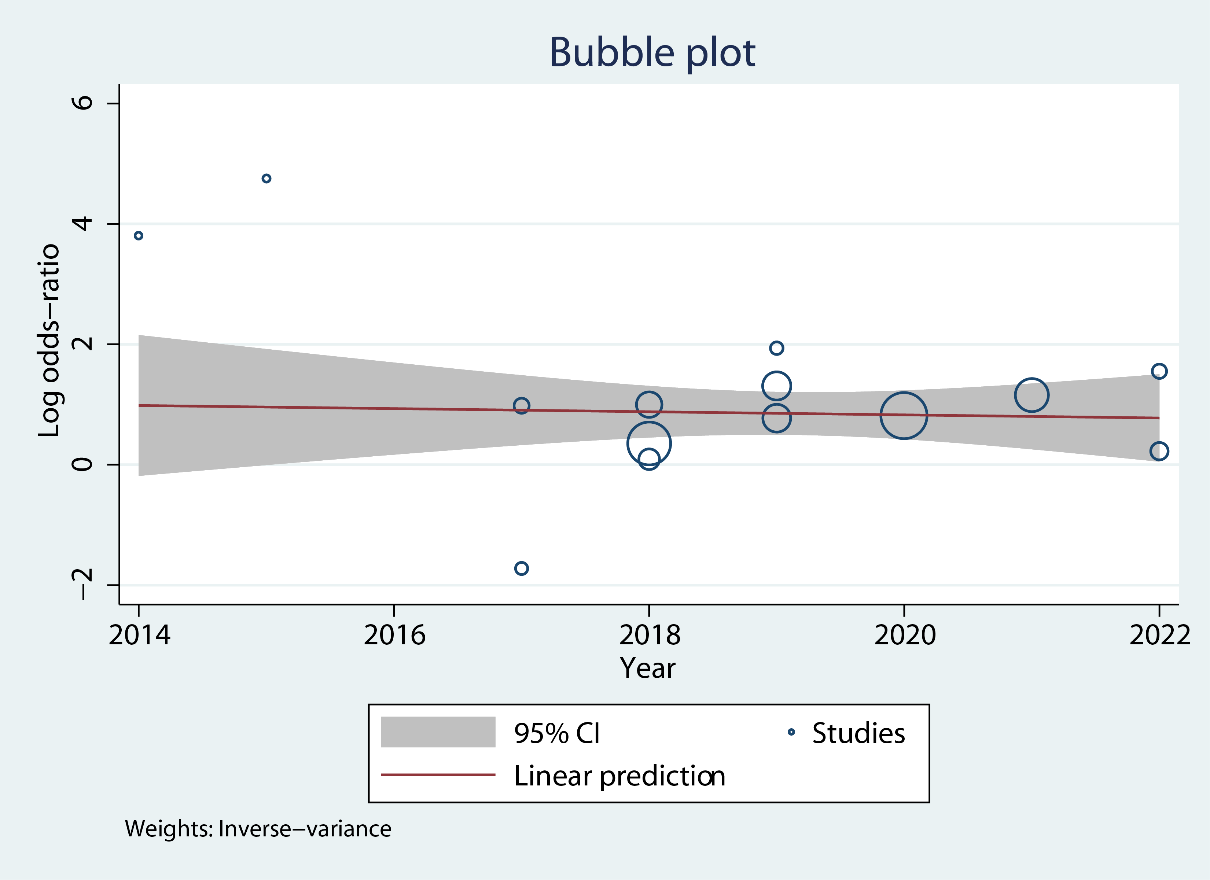
**

**Fig S8. Sensitivity analysis of the 6-month primary patency rate.**

**
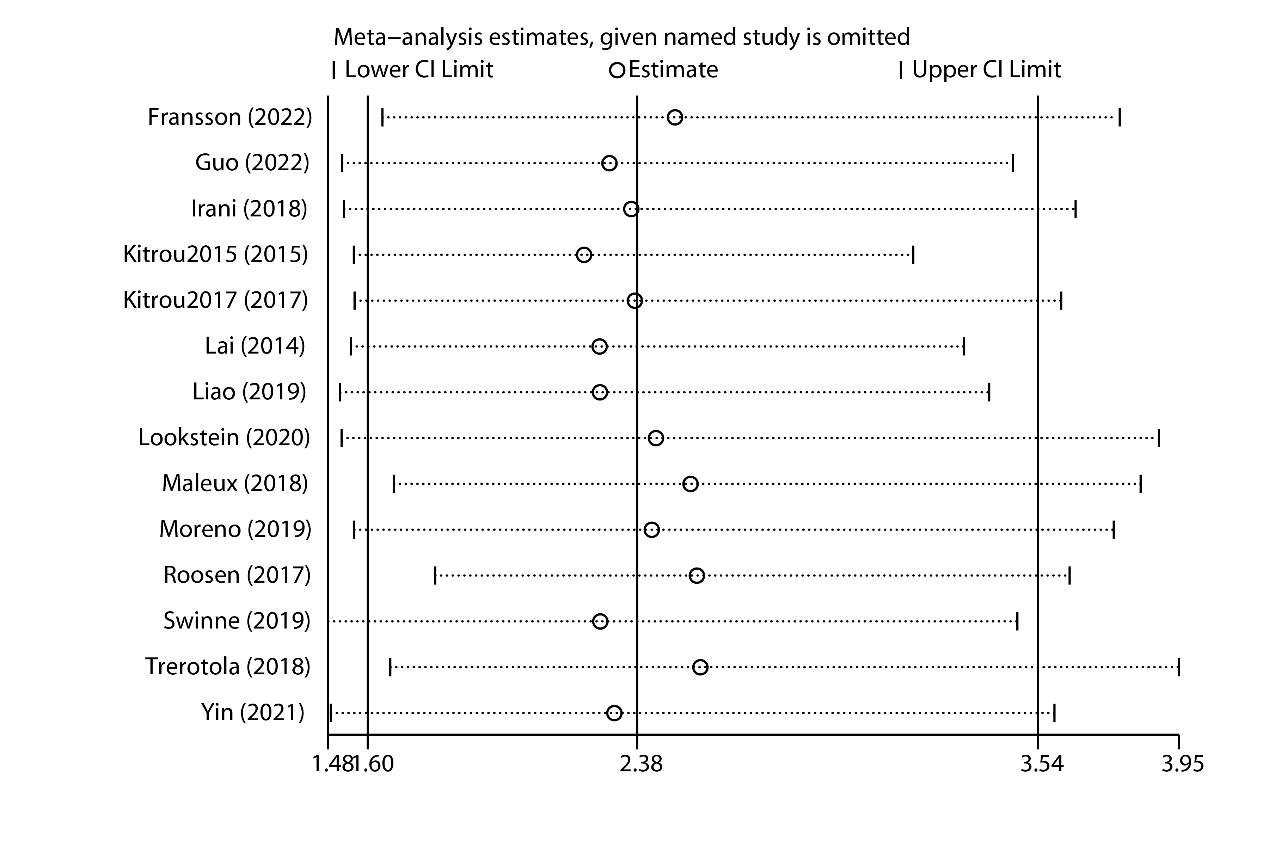
**

**Fig S9. Galbraith plot of the 6-month primary patency rate.**

**
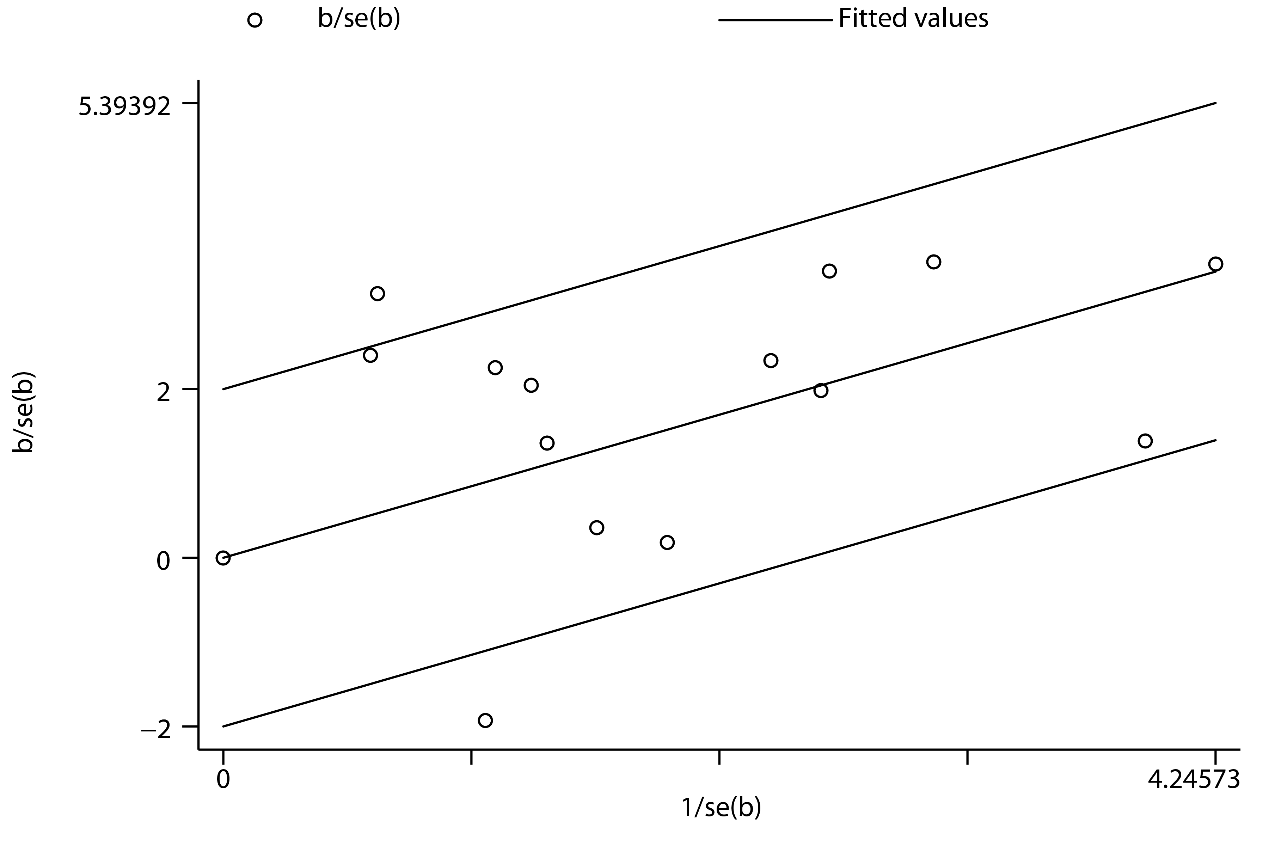
**

**Fig S10. Begg' s plots of the 6-month primary patency rate.**

**
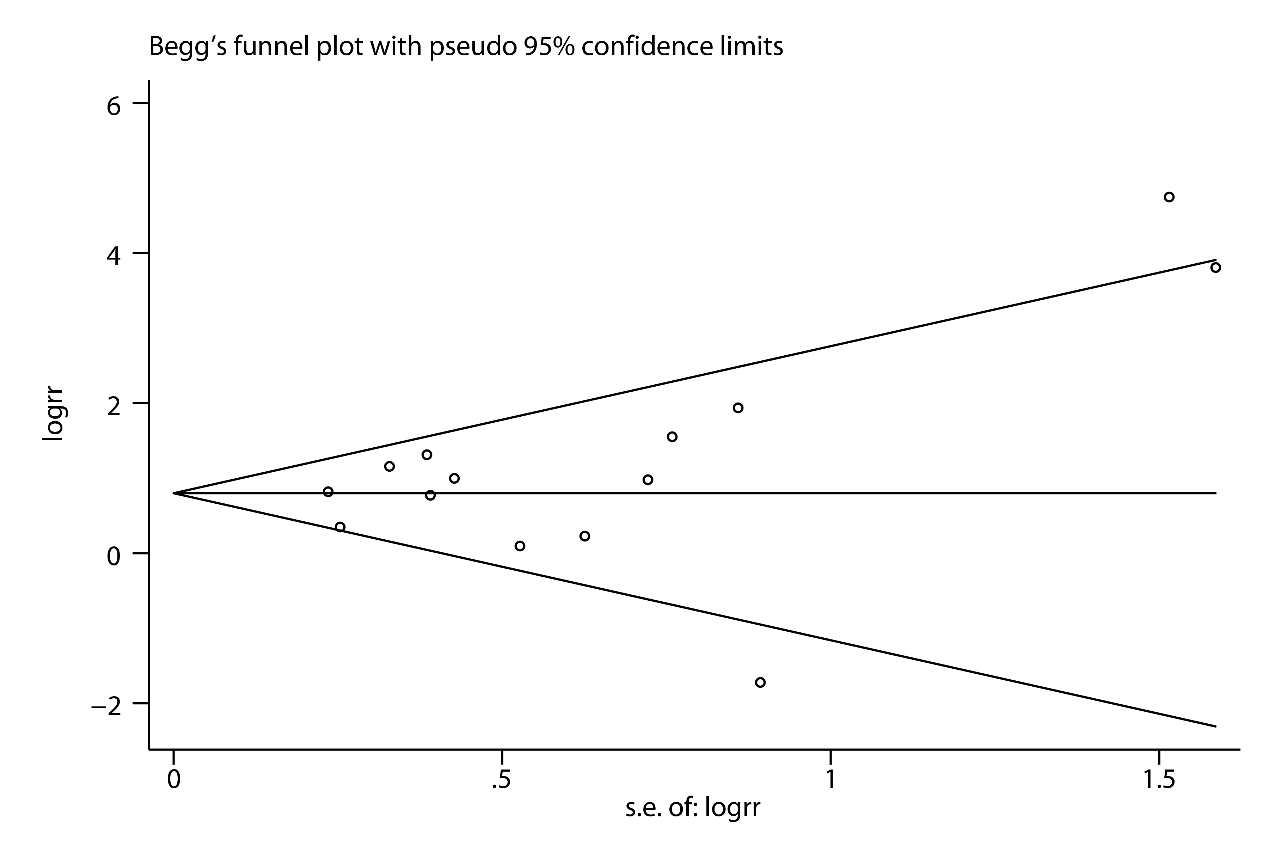
**

**Fig S11. Egger's plots of the 6-month primary patency rate.**

**
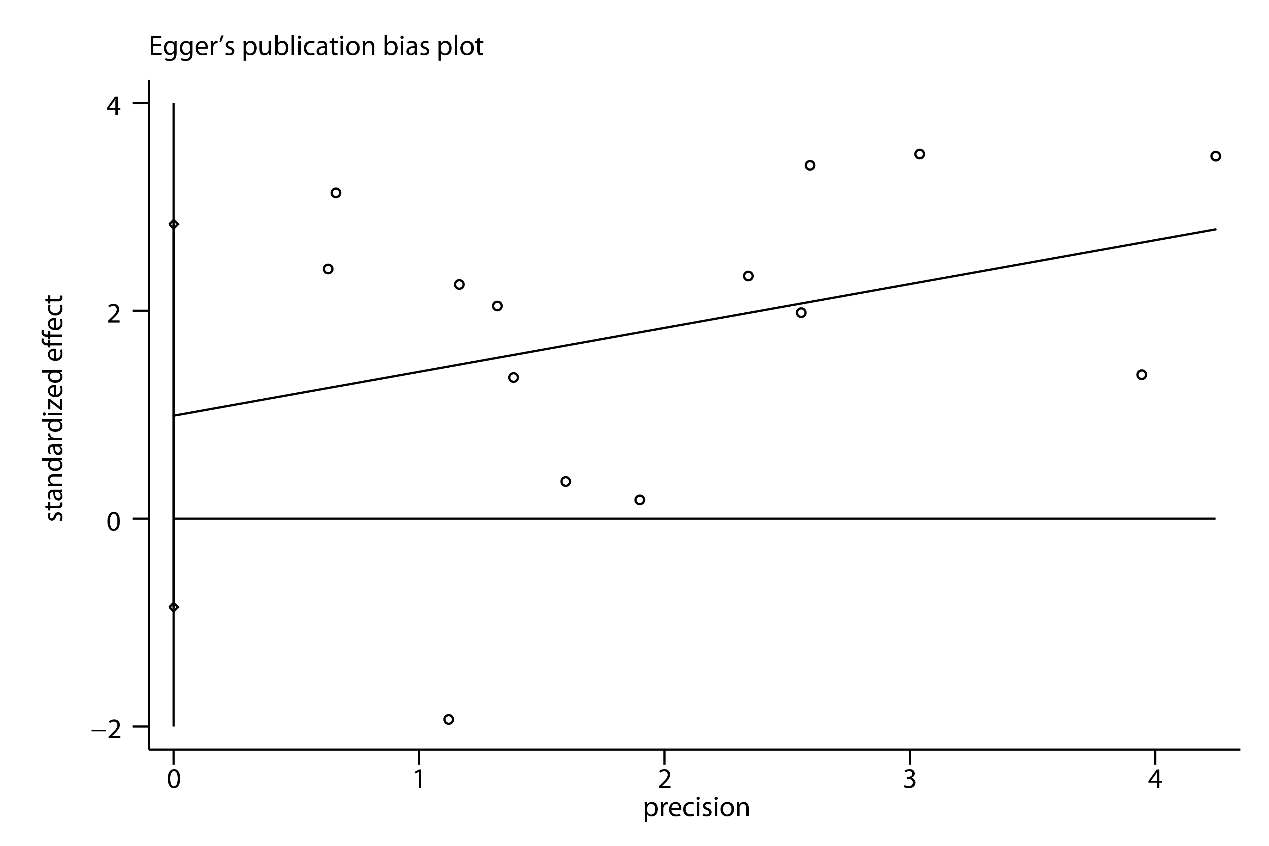
**

**Fig S12. Funnel plot of the 6-month primary patency rate.**

**
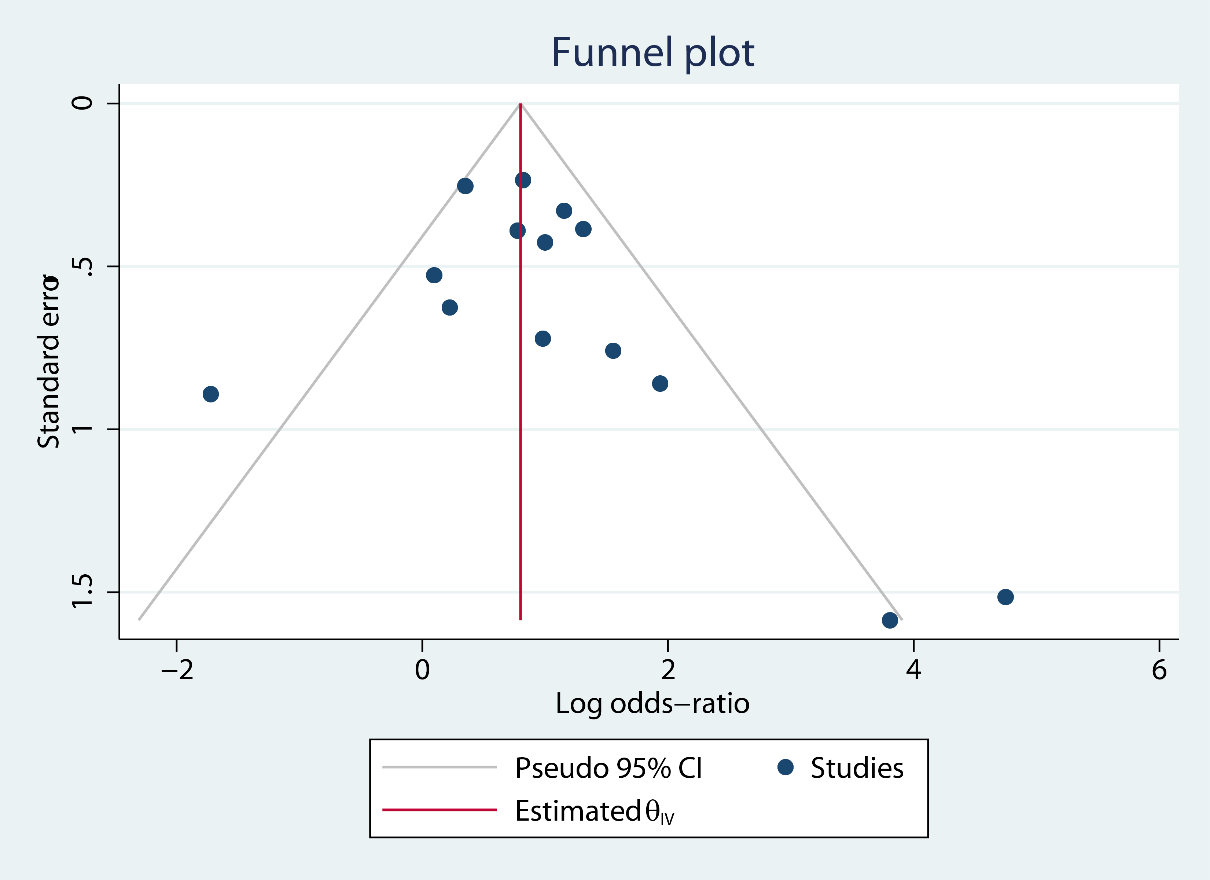
**

**Fig S13. Forest plot of 12-month primary patency of target lesion.**

**
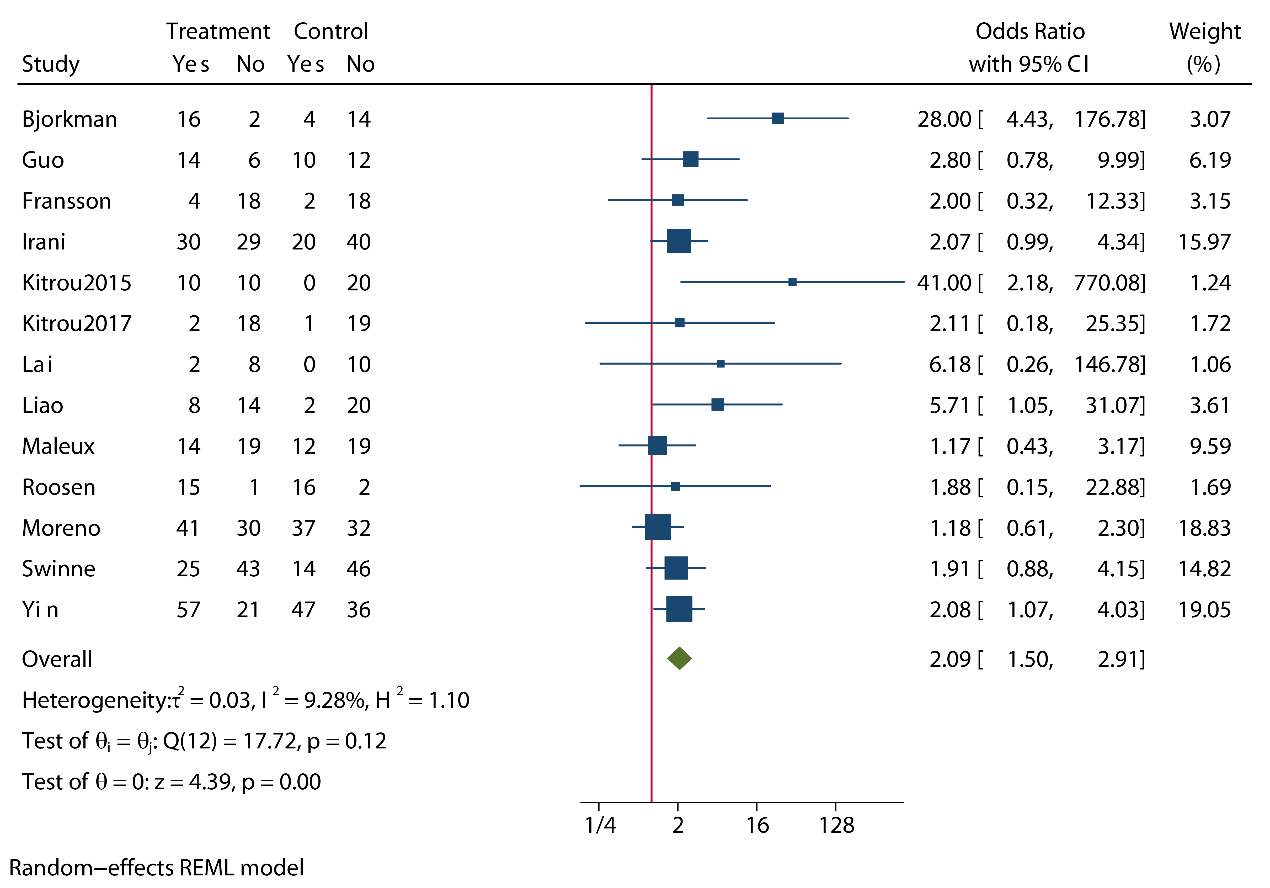
**

**Fig S14. L'Abbe plot of the 12-month primary patency rate.**

**
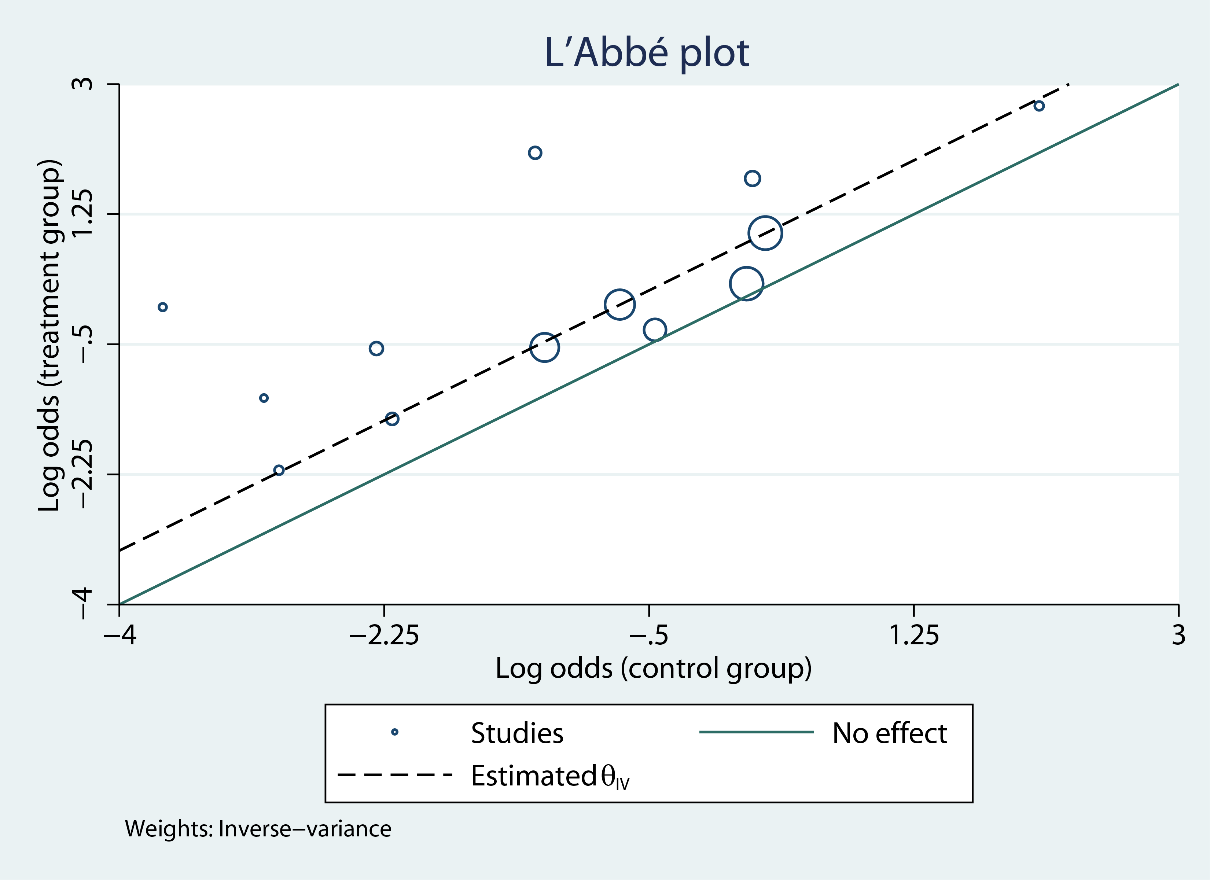
**

**Fig S15. Subgroup analysis of the 6-month primary patency rate.**

**
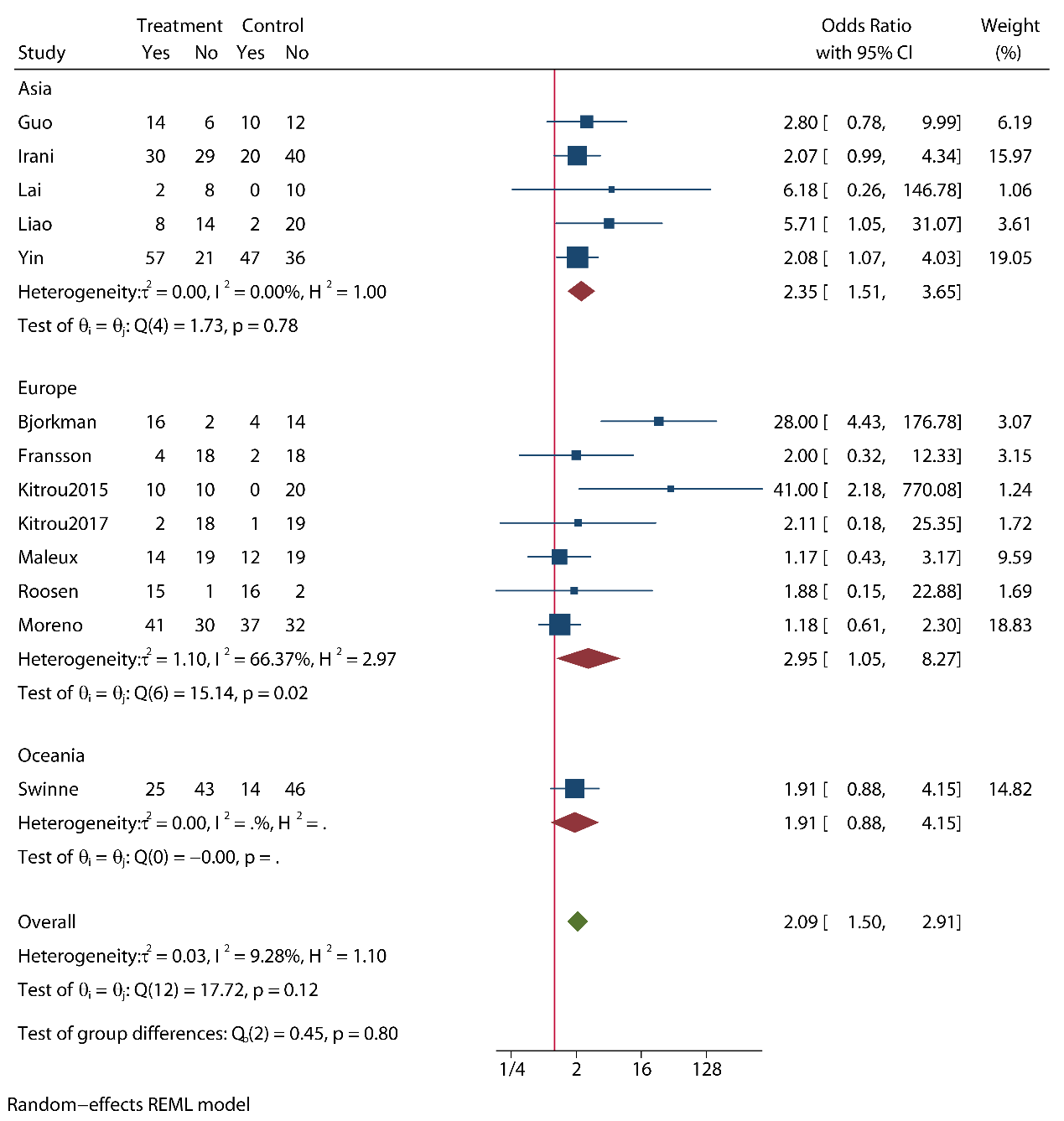
**

**Fig S16. Meta-regression of study size of 12-month primary patency rate.**

**
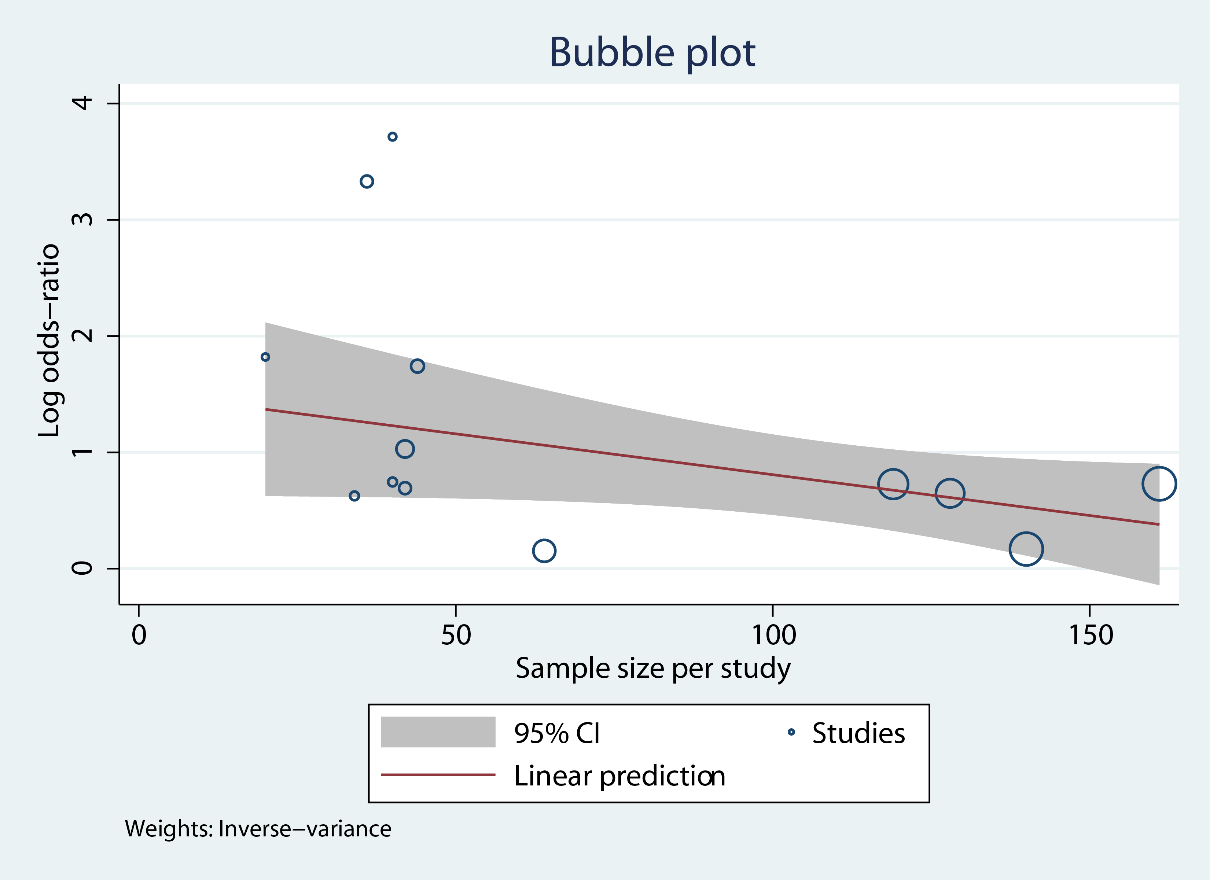
**

**Fig S17. Meta-regression of publication year of 12-month primary patency rate.**

**
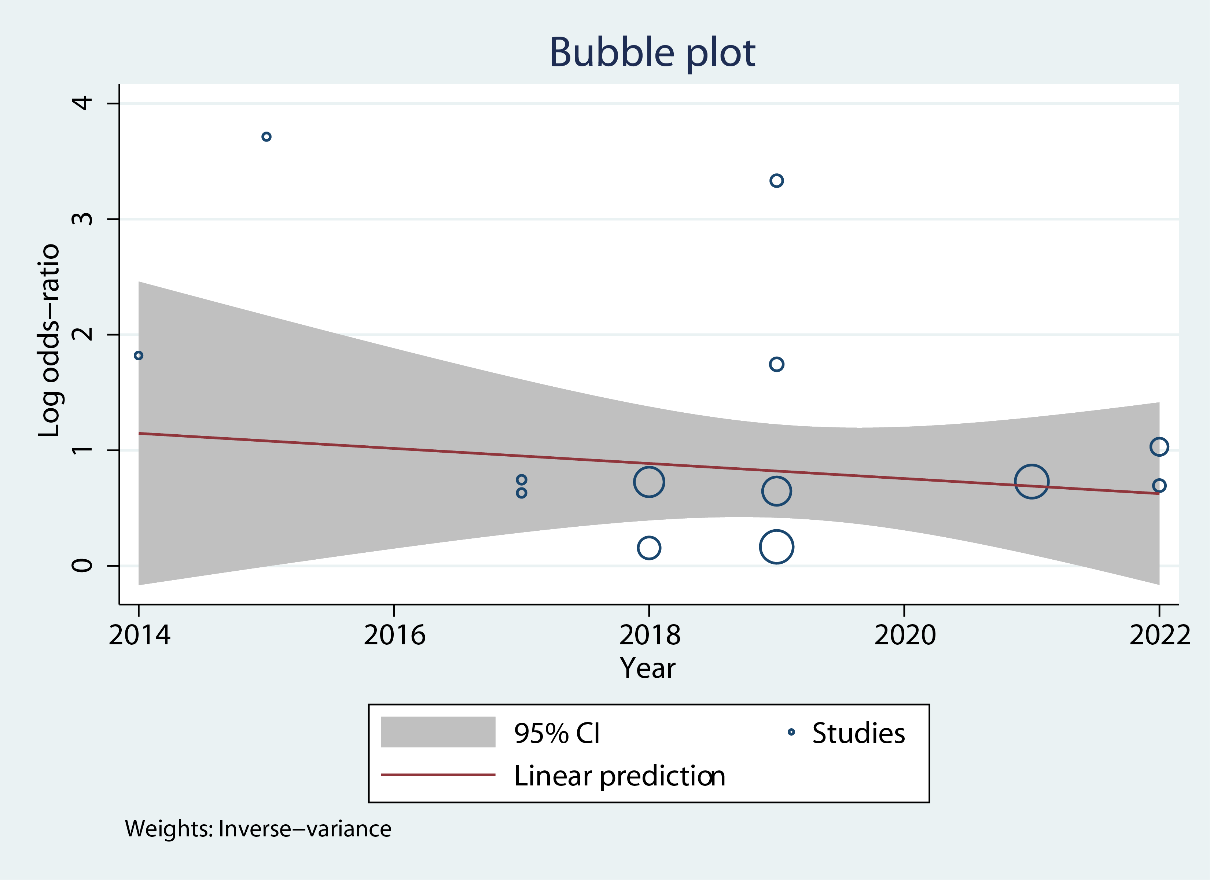
**

**Fig S18. Sensitivity analysis of the 12-month primary patency rate.**

**
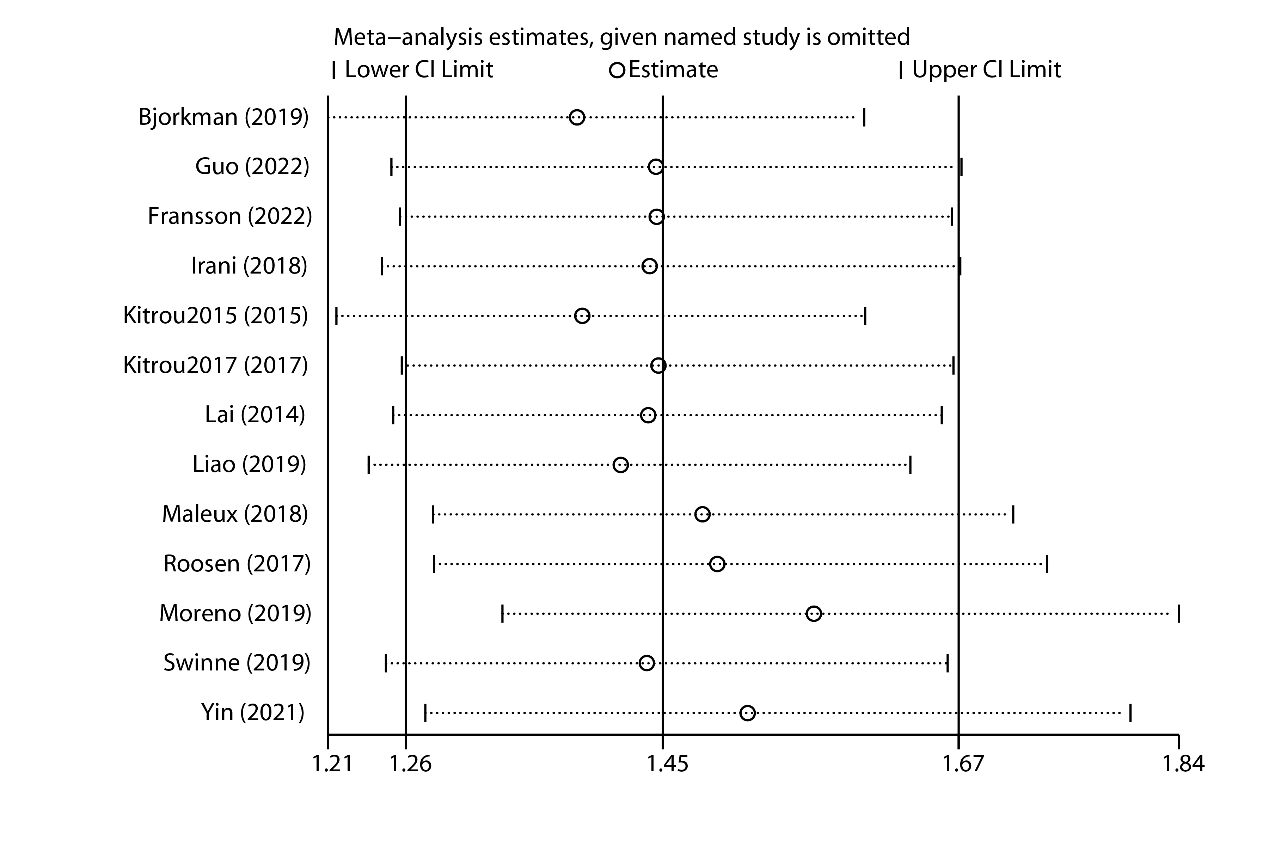
**

**Fig S19. Galbraith plot of the 12-month primary patency rate.**

**
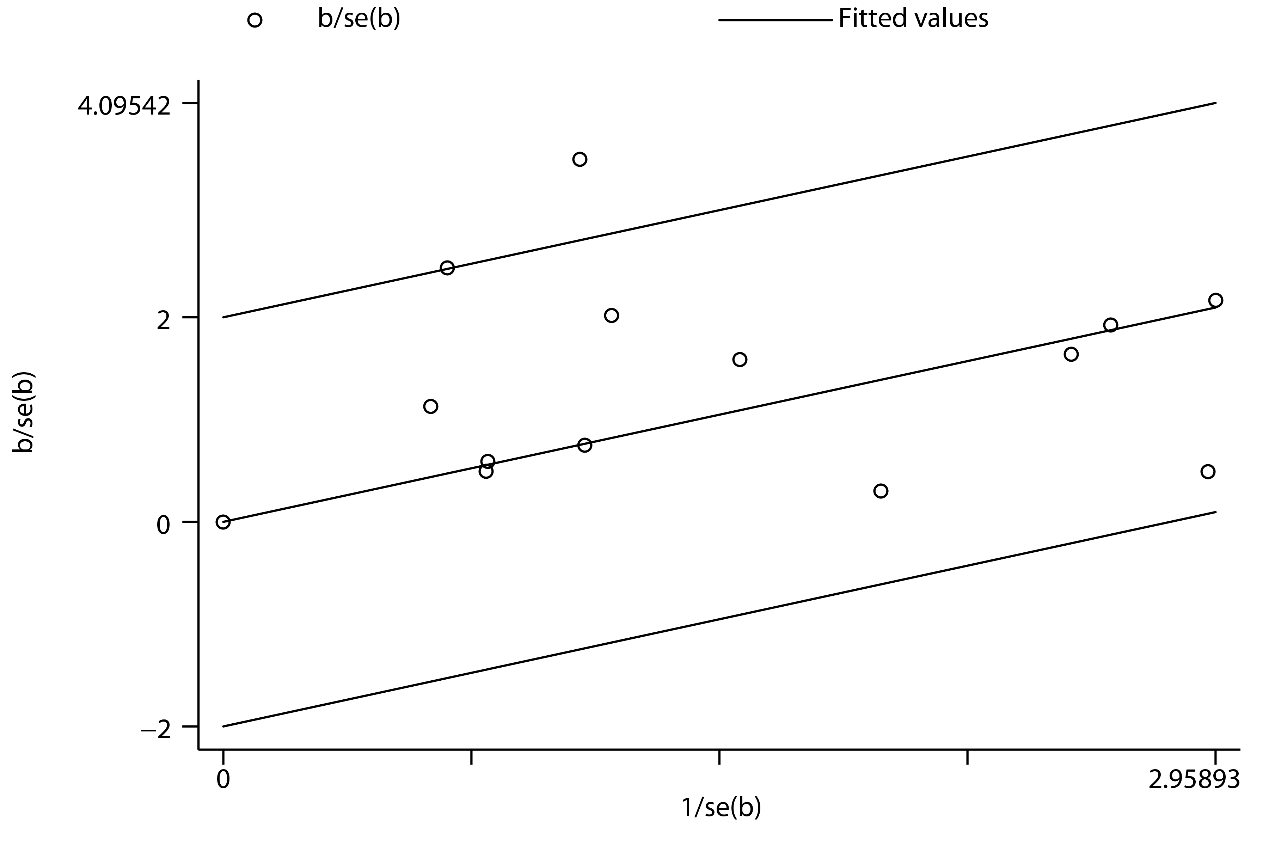
**

**Fig S20. Begg' s plots of the 12-month primary patency rate.**

**
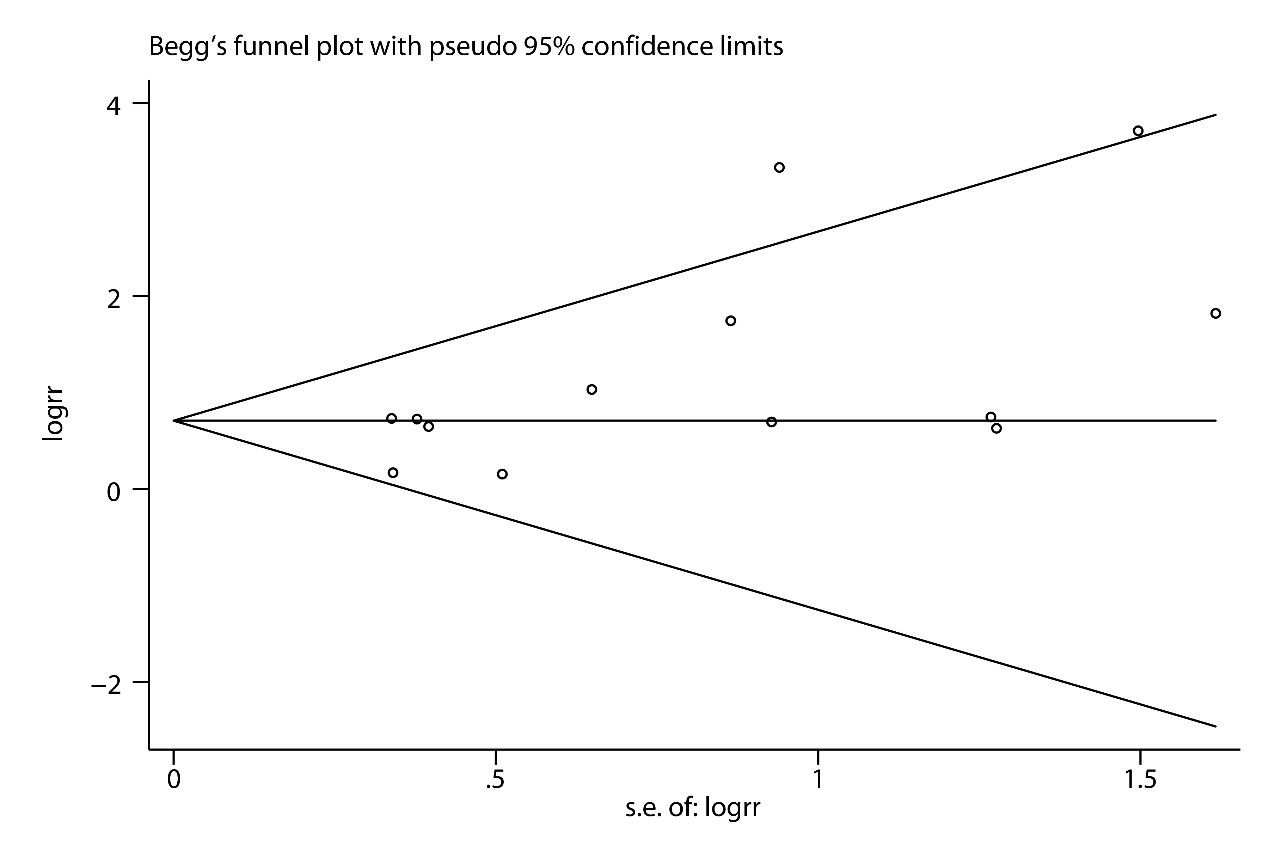
**

**Fig S21. Egger' s plots of the 12-month primary patency rate.**

**
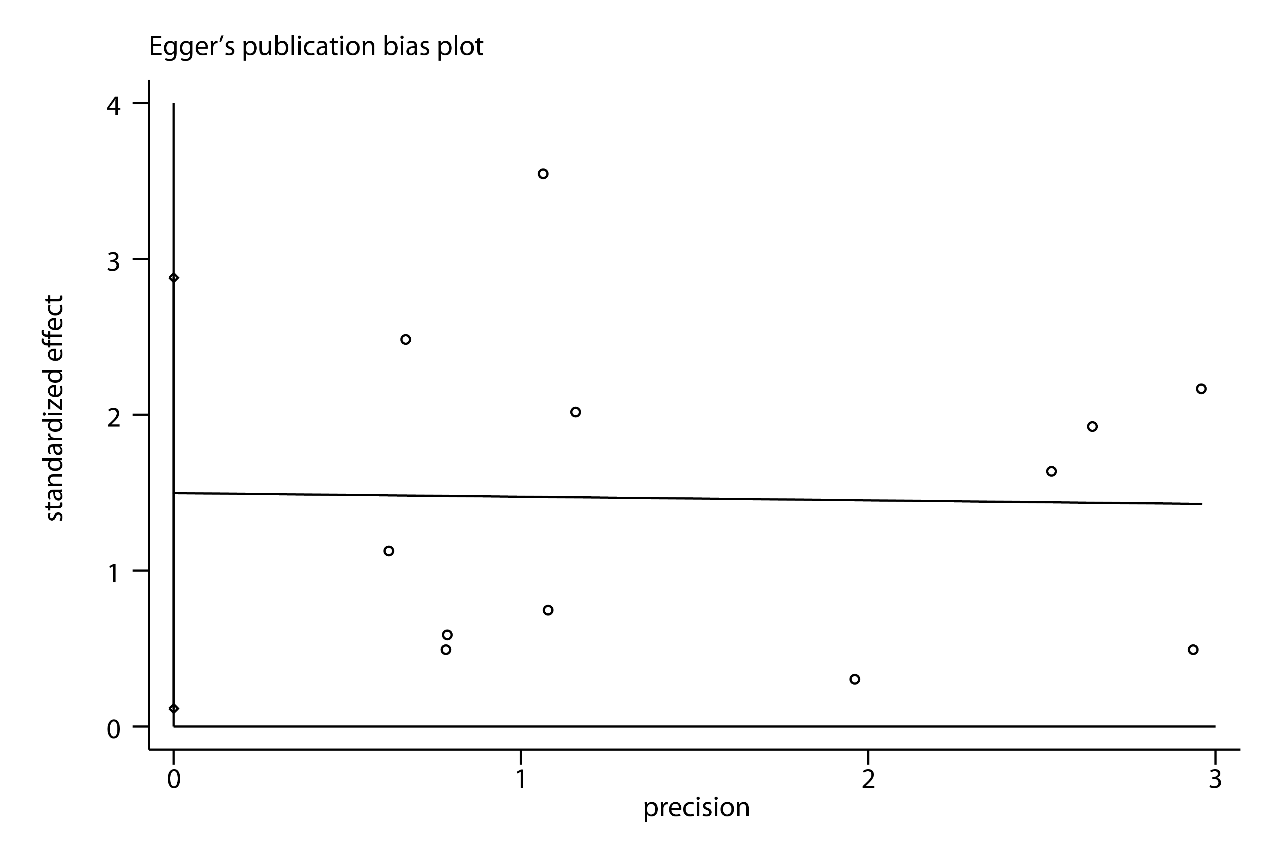
**

**Fig S22. Funnel plot of 12-month primary patency of target lesion.**

**
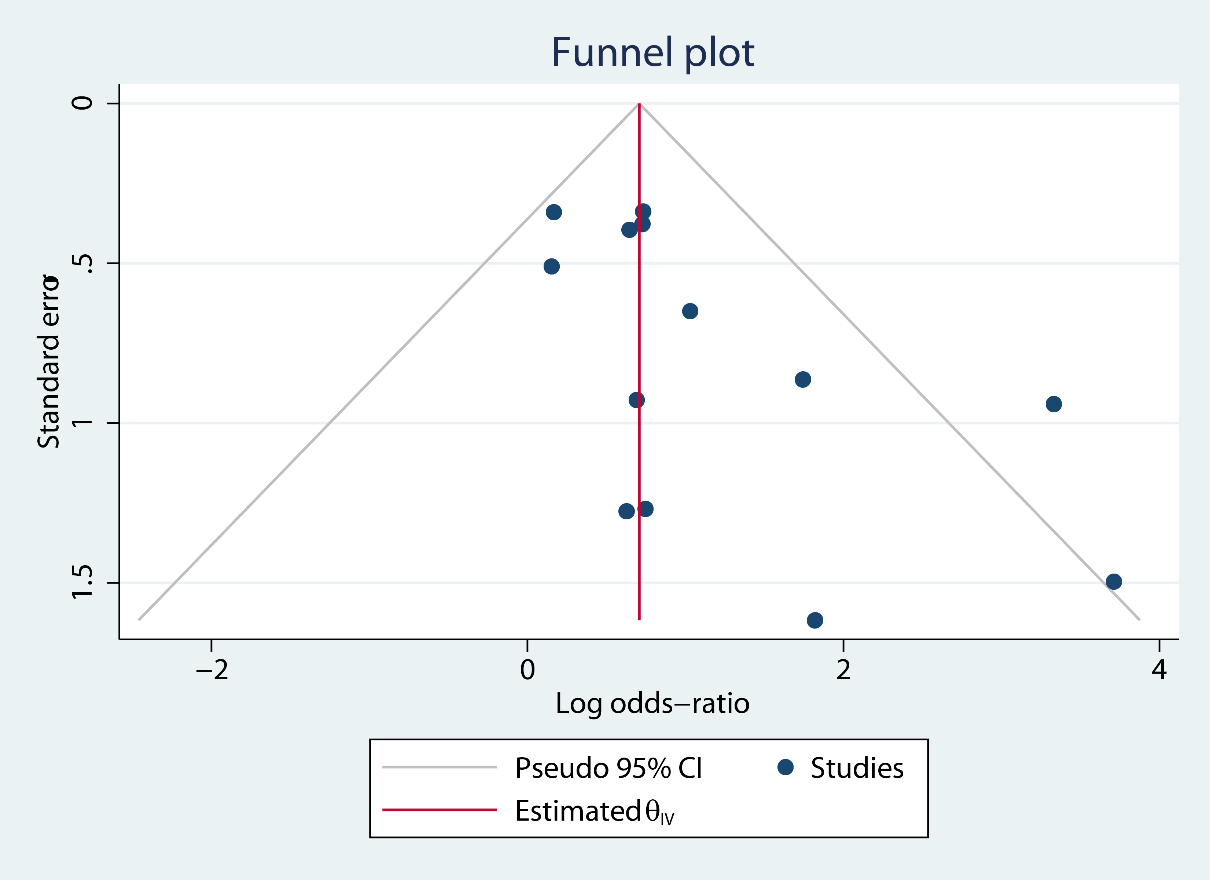
**

**Fig S23. Meta-regression of sample size of all-cause mortality at 6 months.**

**
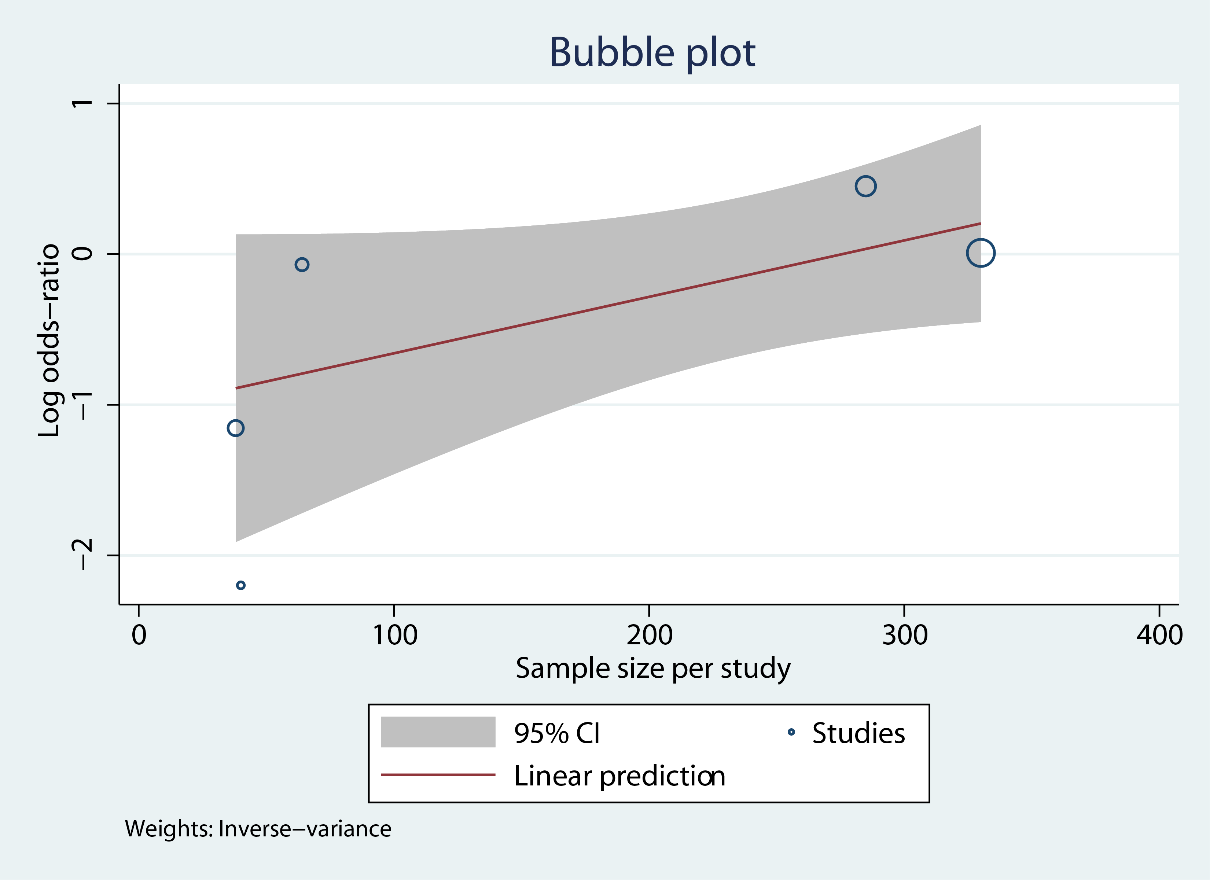
**

**Fig S24. Meta-regression of publication year of all-cause mortality at 6 months.**

**
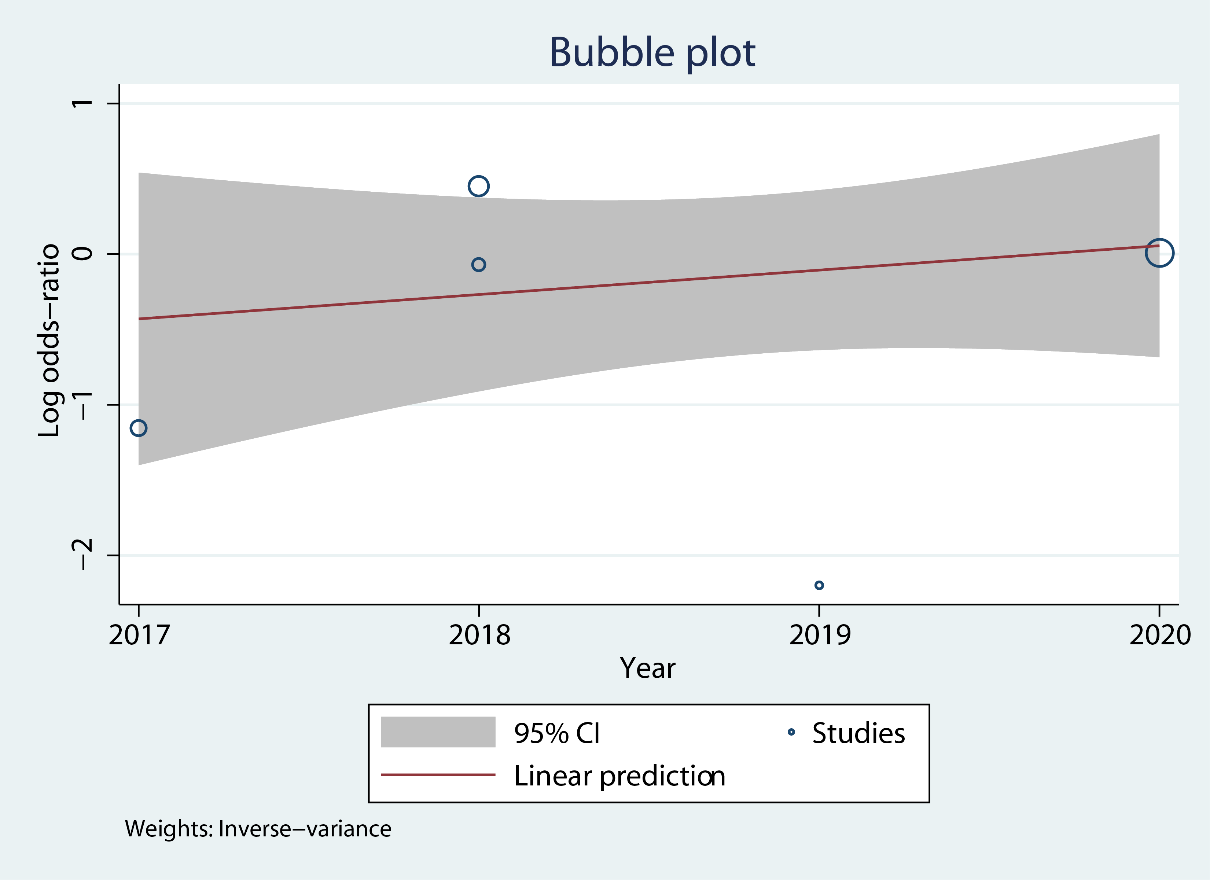
**

**Fig S25. Sensitivity analysis of all-cause mortality at 6 months.**

**
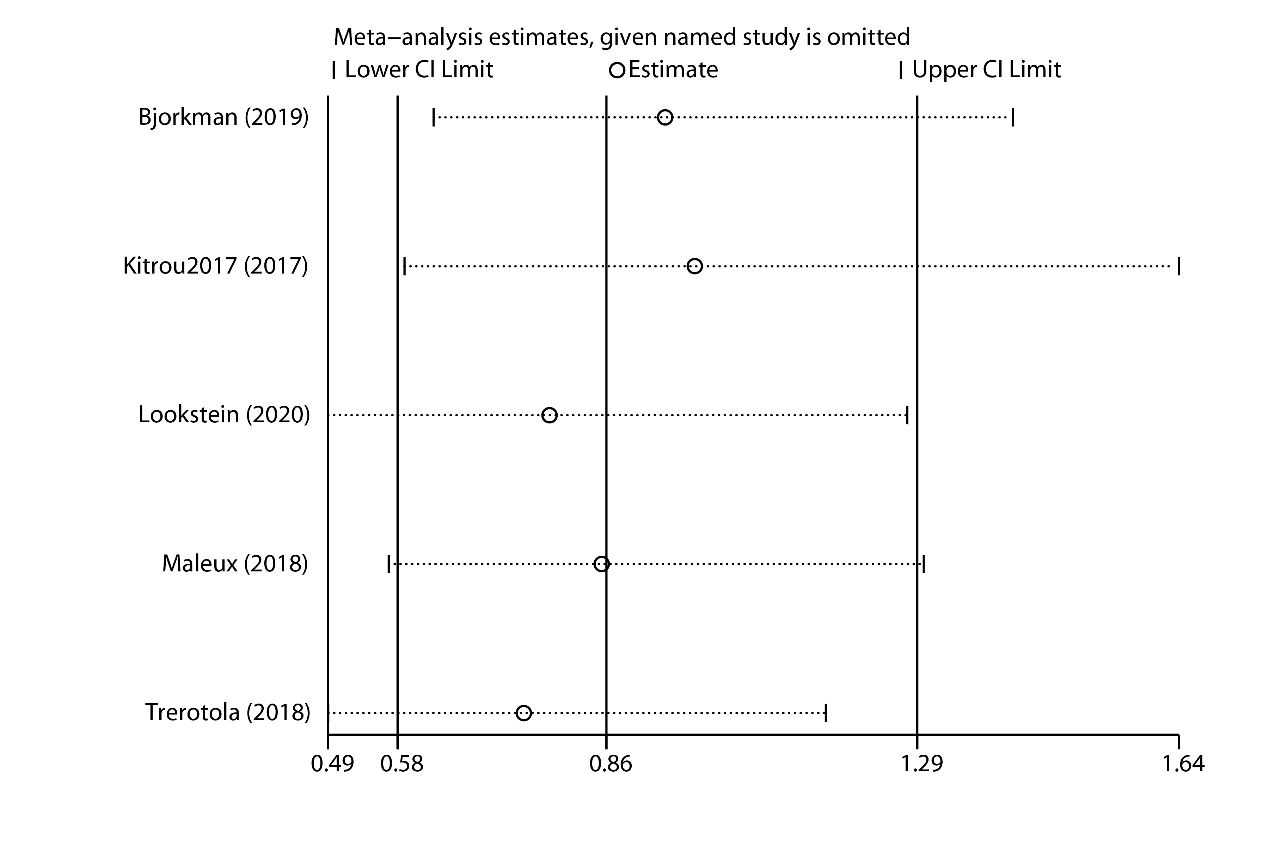
**

**Fig S26. Galbraith plot of all-cause mortality at 6 months.**

**
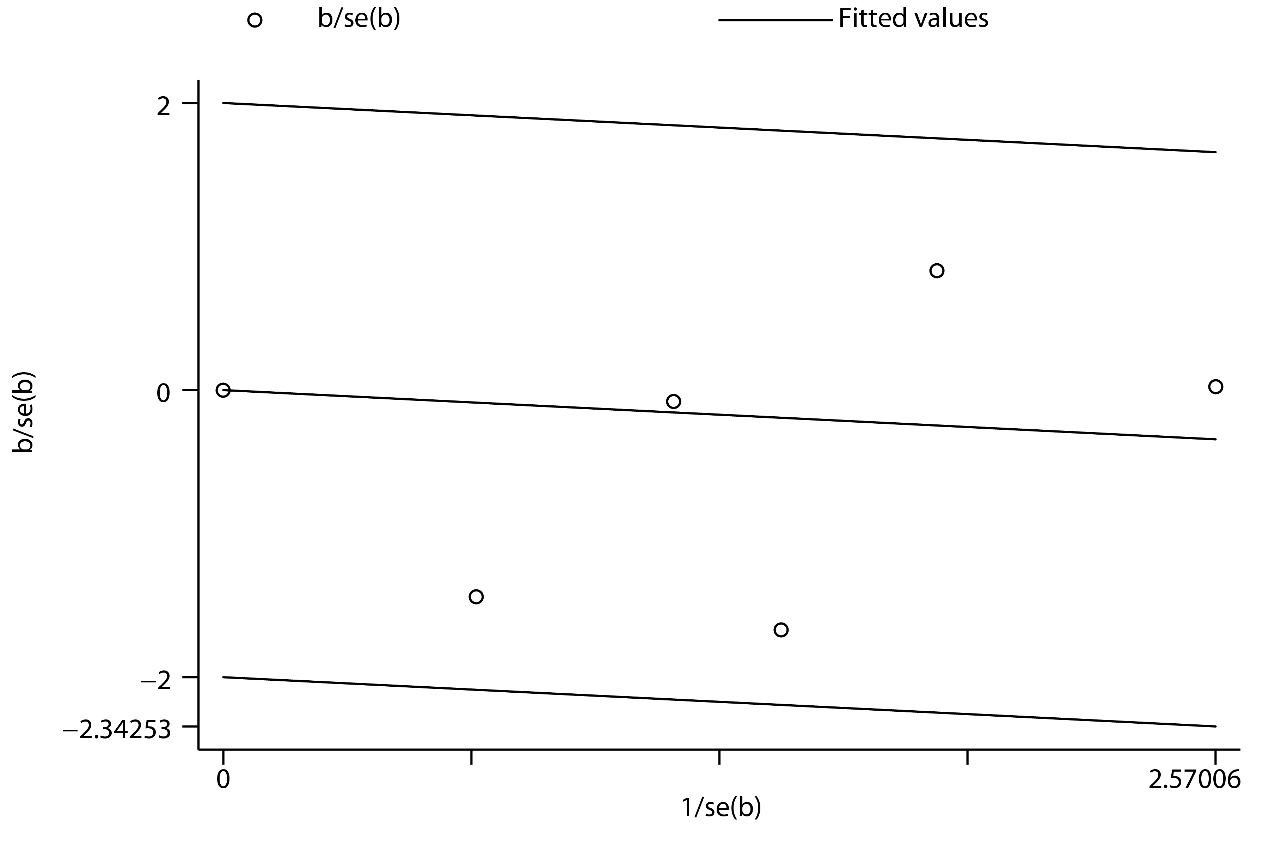
**

**Fig S27. Begg's plots of all-cause mortality at 6 months.**

**
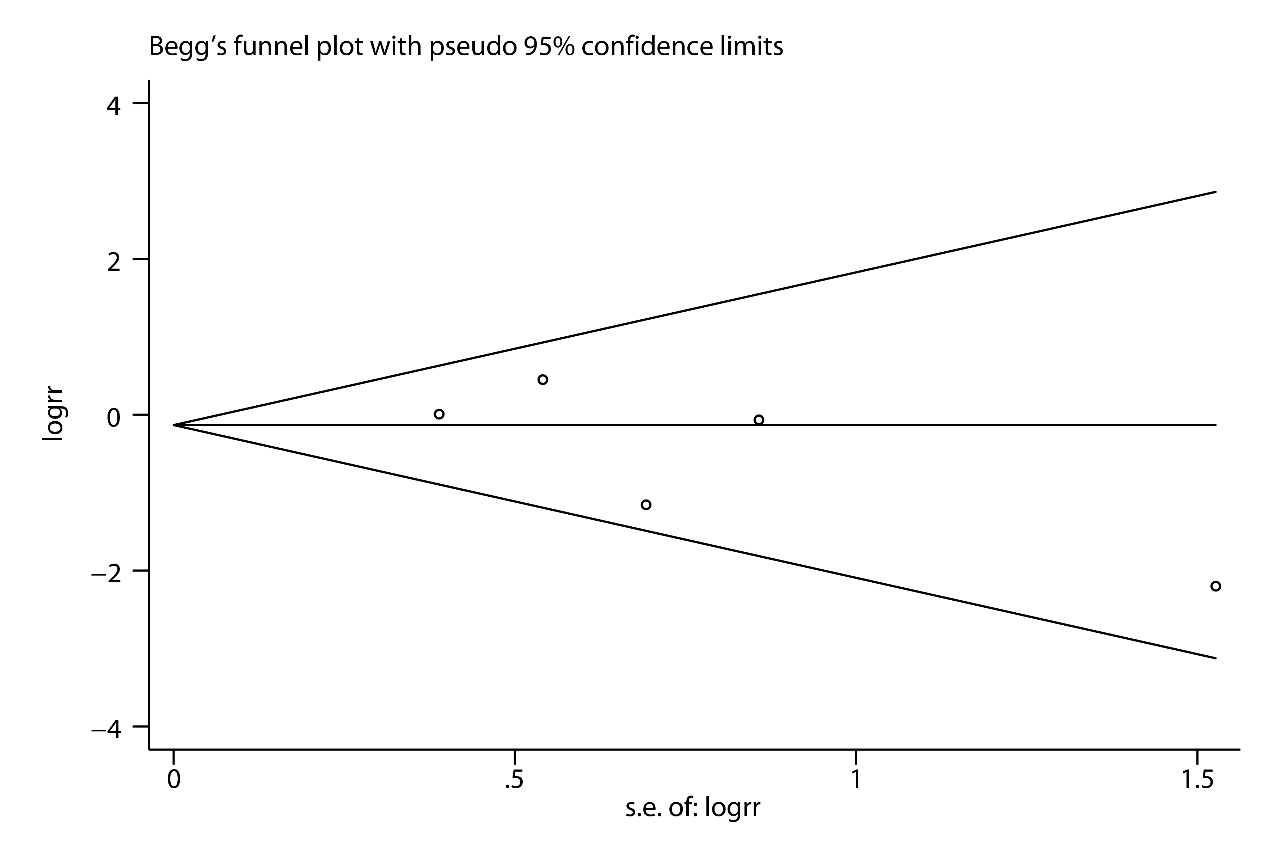
**

**Fig S28. Egger's plots of all-cause mortality at 6 months.**

**
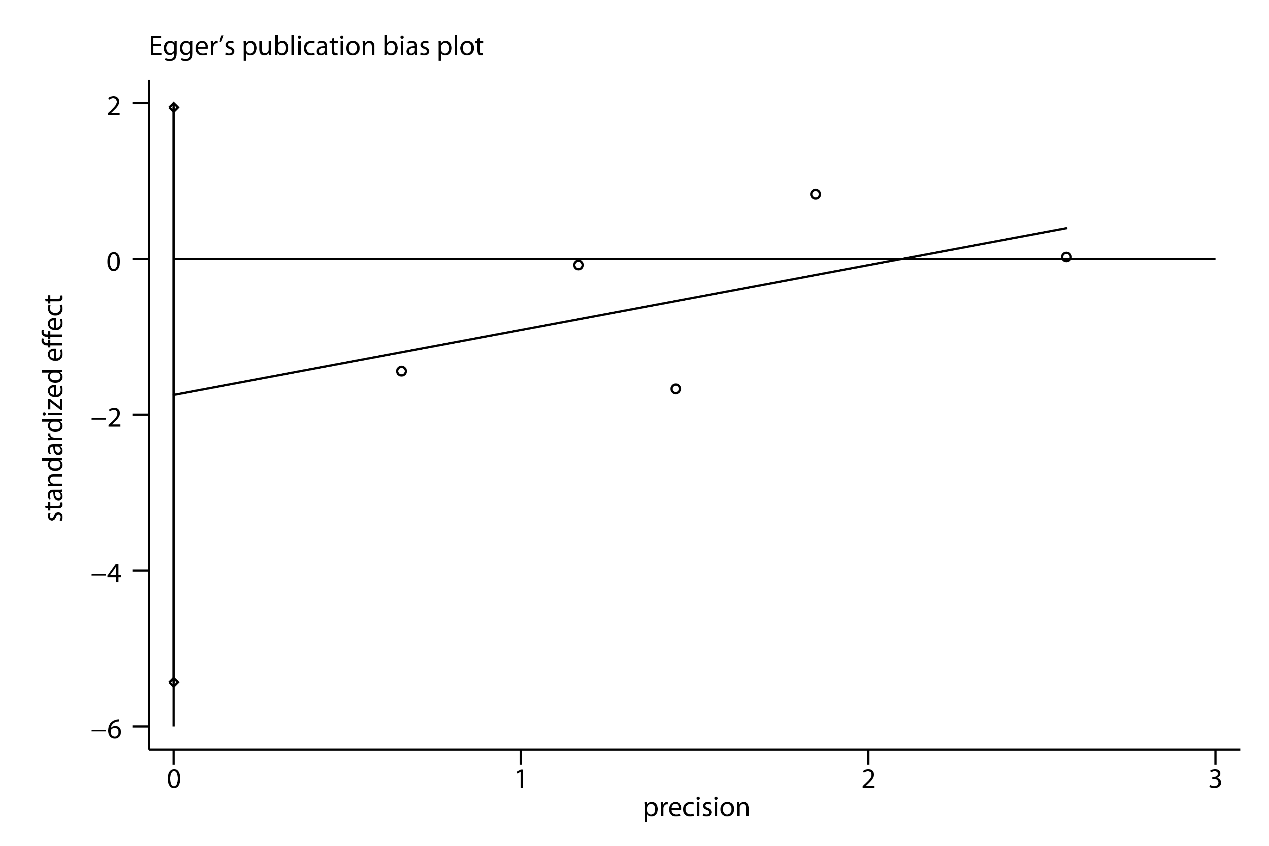
**

**Fig S29. Funnel plot of all-cause mortality at 6 months.**

**
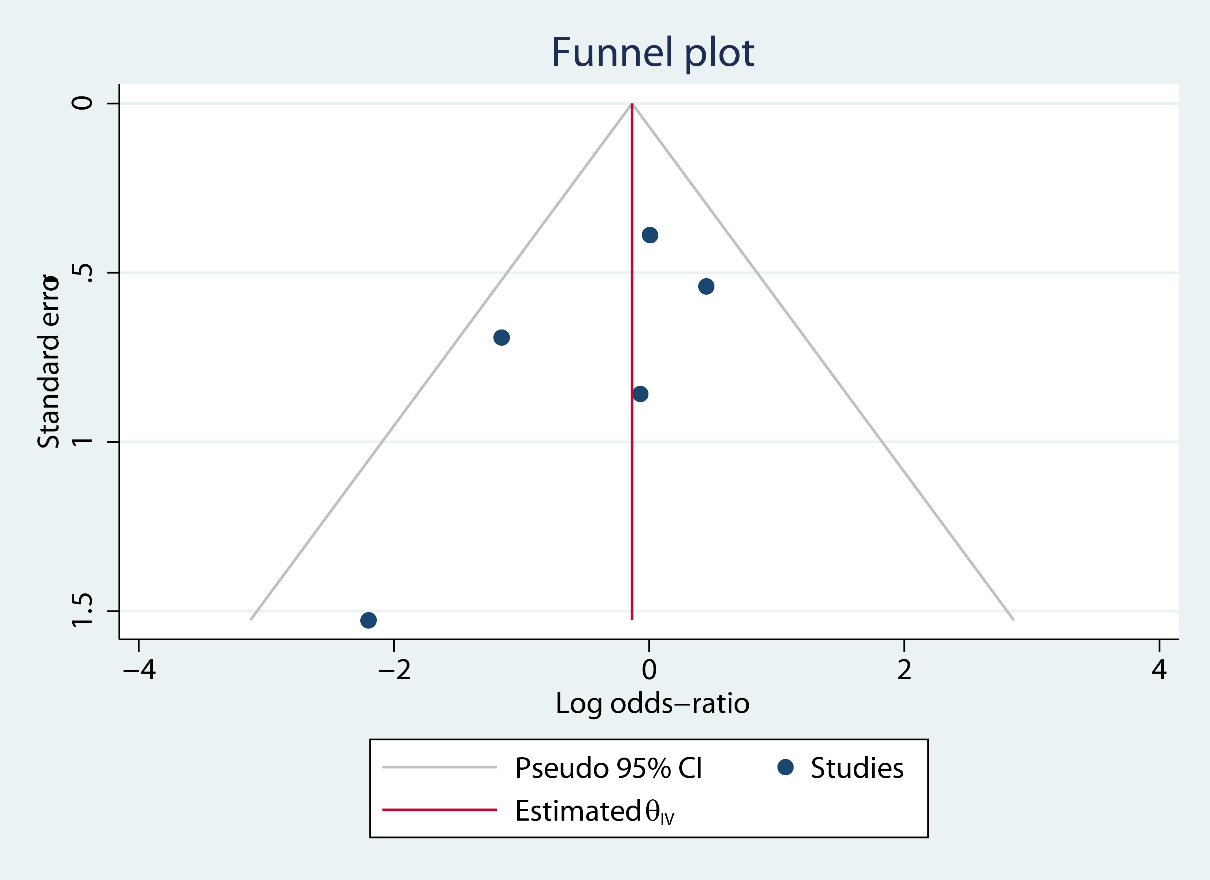
**

**Fig S30. Meta-regression of sample size of all-cause mortality at 12-months.**

**
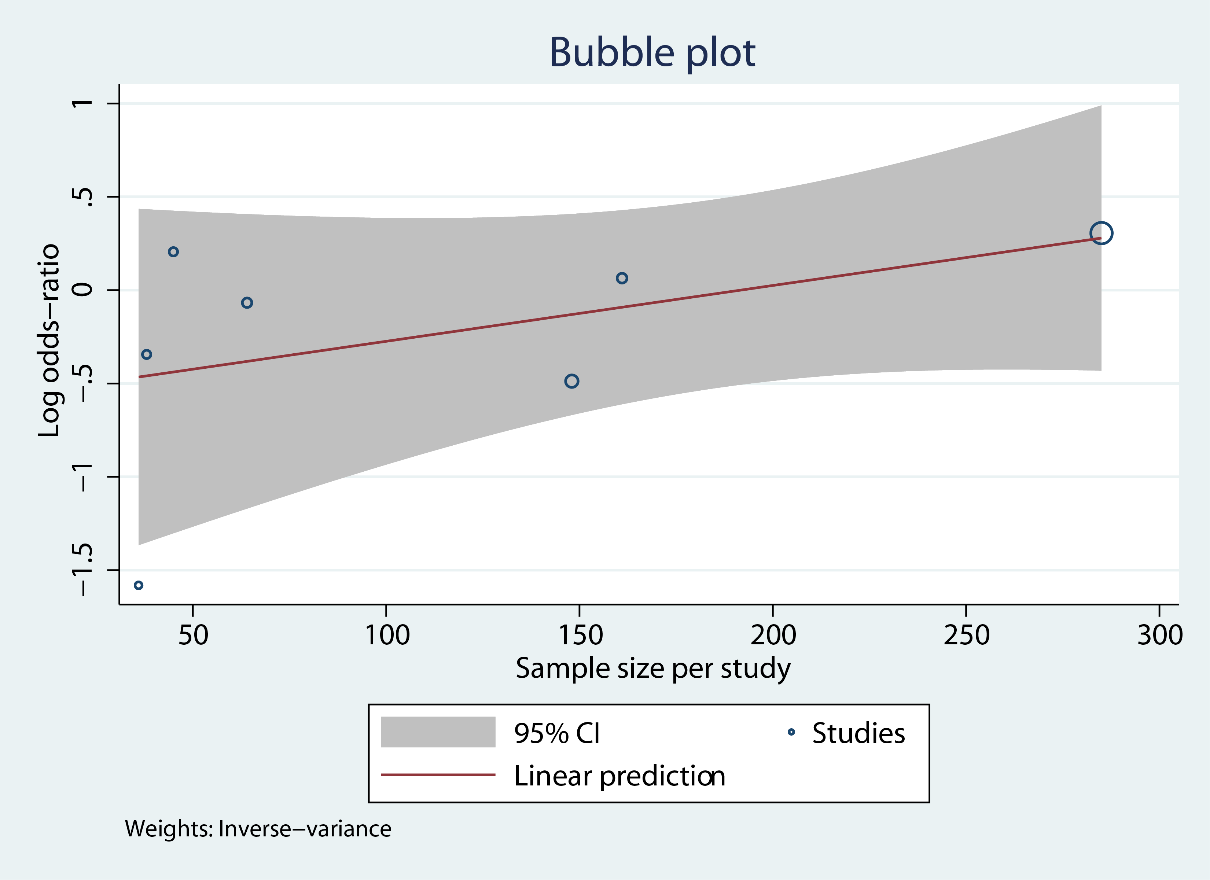
**

**Fig S31. Meta-regression of publication year of all-cause mortality at 12-months.**

**
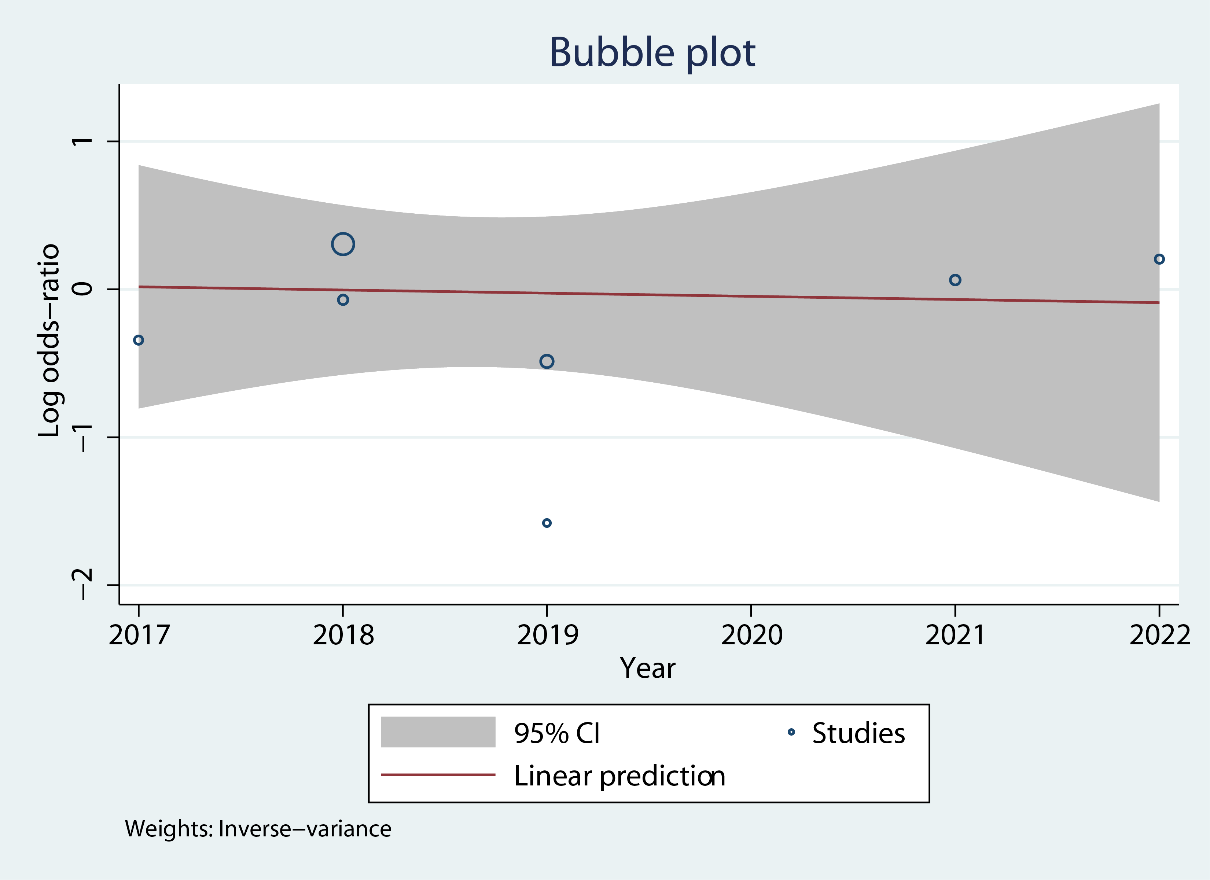
**

**Fig S32. Sensitivity analysis of all-cause mortality at 12 months.**

**
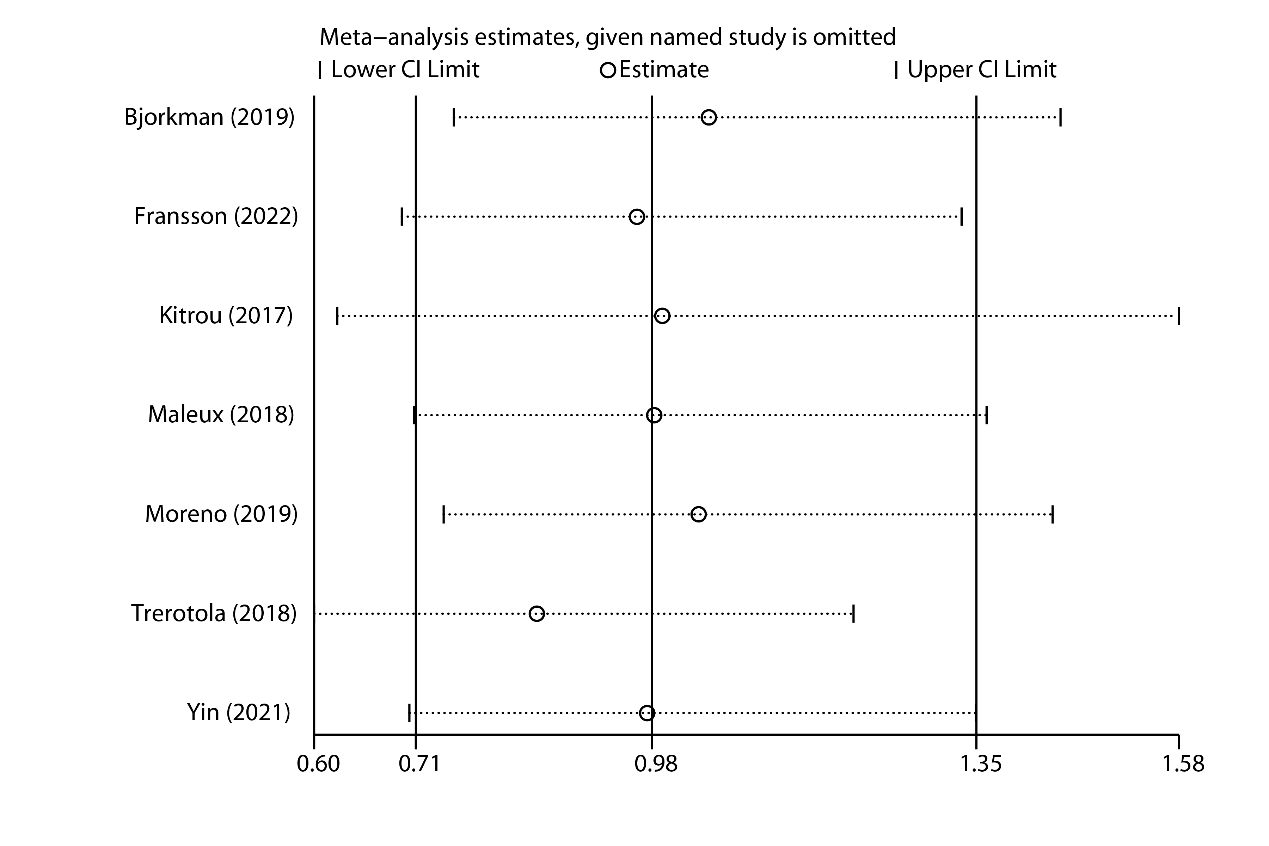
**

**Fig S33. Galbraith plot of all-cause mortality at 12 months.**

**
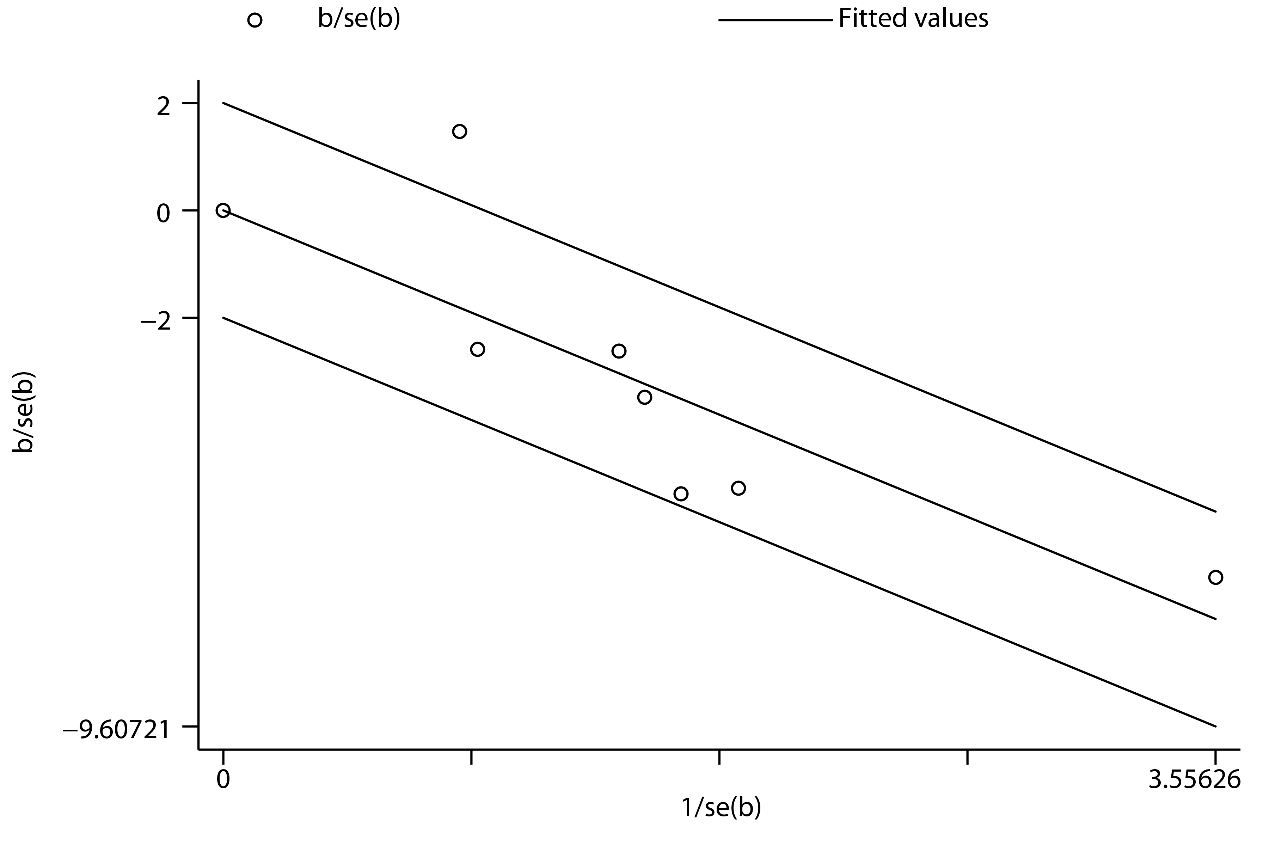
**

**Fig S34. Begg's plots of all-cause mortality at 12 months.**

**
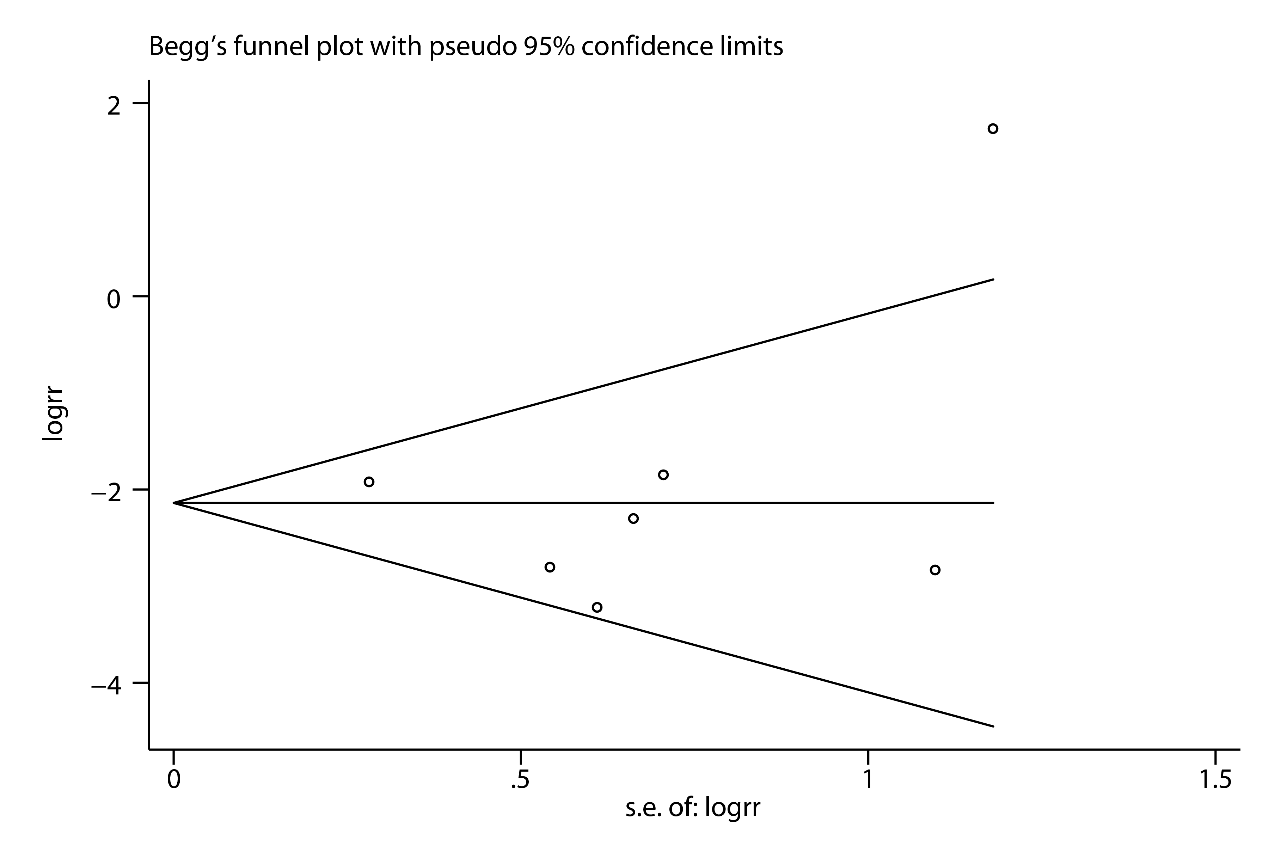
**

**Fig S35. Egger's plots of all-cause mortality at 12 months.**

**
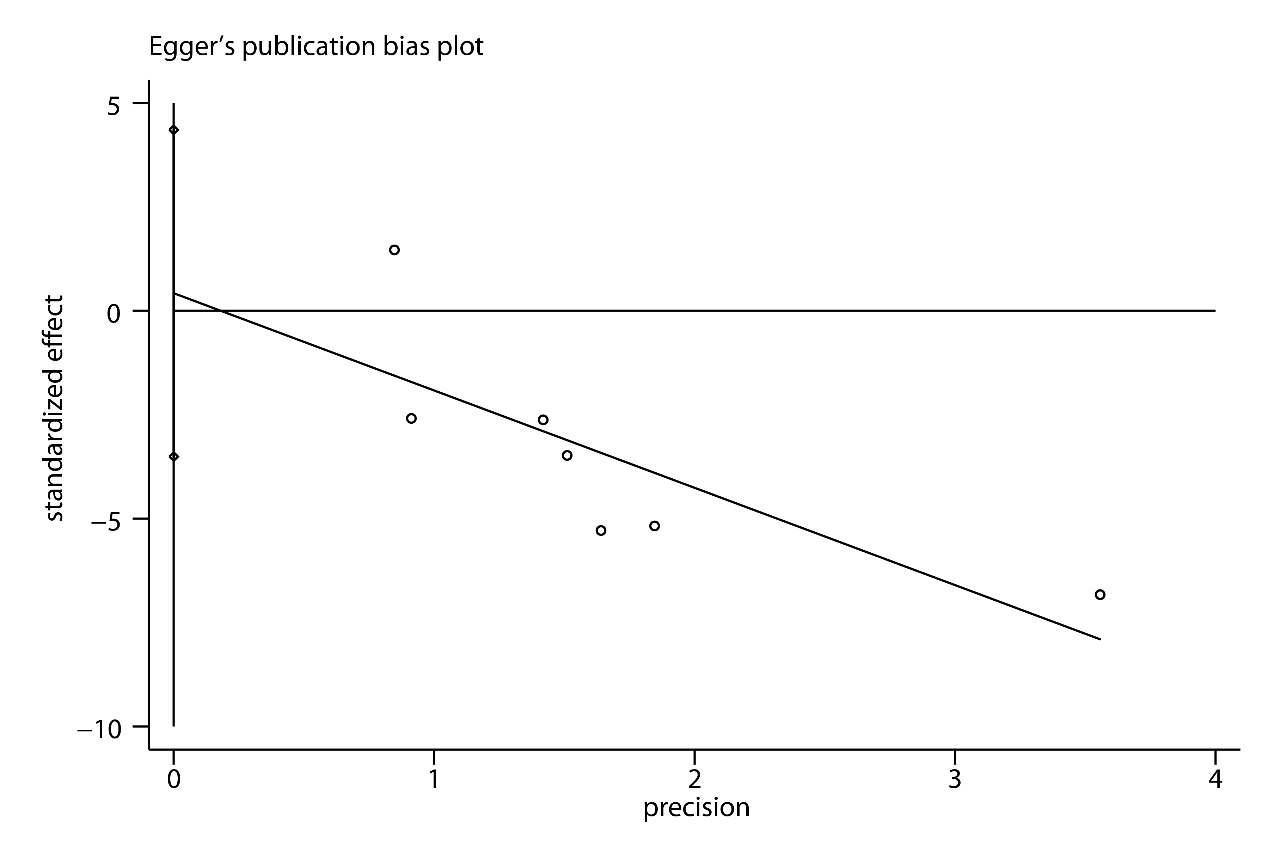
**

**Fig S36. Funnel plot of all-cause mortality at 12 months.**


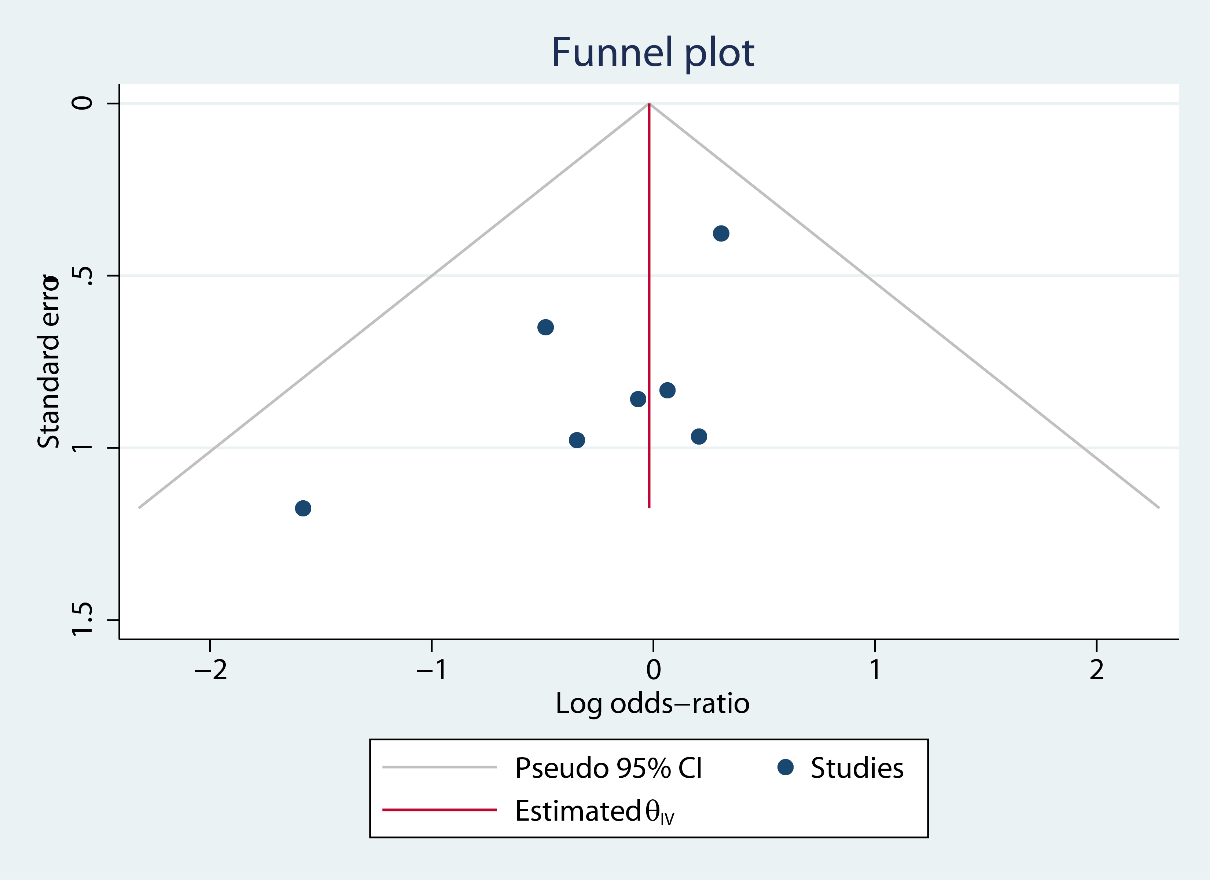

Supplement: Supplementary file 1 — Supporting information. [file CLC-46-877-s001.doc]
