# Supplementary material for: Comparison of drug‐coated balloon angioplasty versus common balloon angioplasty for arteriovenous fistula stenosis: A systematic review and meta‐analysis
Source: Clin Cardiol. 2023 Jul 7;46(8):877–85. doi: 10.1002/clc.24078 (PMC10436783; doi:10.1002/clc.24078)
Supplement: Supplementary file 2 — Supporting information. [file CLC-46-877-s002.doc]

**Table S1. Main characteristics of the included RCTs in comorbidities and hemodialysis vascular access.**

| Study | Hypertension | |  | Diabetes mellitus | |  | Length of  stenosis | |  | Duration of fistula use | |  | Antiplatelet  therapy | |
| --- | --- | --- | --- | --- | --- | --- | --- | --- | --- | --- | --- | --- | --- | --- |
|  | DCB | CB |  | DCB | CB |  | DCB | CB |  | DCB | CB |  | DCB | CB |
| Bjorkman2019 | 14(44) | 16(44) |  | 11(33) | 11(44) |  | N | N |  | 2.4(1.6–3.6) | 13(4–10) |  | 3 (16.7) | 1(5.6) |
| Fransson2022 | 17(77) | 16(80) |  | 11 (50) | 9 (45) |  | N | N |  | 17 (77) | 11 (55) |  | 12 (60) | 12 (55) |
| Guo2022 | 3 (15) | 3 (14) |  | 7 (35) | 9 (41) |  | N | N |  | 22.6±5.9 | 22.0±7.4 |  | 12 | N |
| Irani2018 | 55 (93.2) | 55 (91.7) |  | 37 (62.7) | 34 (56.7) |  | 2.6 | 2.5 |  | 44.4±58.6 | 47.3±54.3 |  | 1.80±2.32 | 2.07±2.74 |
| Kitrou2015 | 3 | 3 |  | 4 | 7 |  | N | N |  | 2.13±2.12 | 2.74±2.4 |  | N | N |
| Kitrou2017 | N | N |  | 13 | 11 |  | 28.4±15.1 | 28.5±18.7 |  | 2.83(1.2–6.1) | 2.42(0.7–4.4) |  | N | N |
| Lai2014 | 4 | 4 |  | 5 | 5 |  | N | N |  | 5.3 ± 2.6 | 5.3 ± 2.6 |  | N | N |
| Liao2019 | 18 (81.8) | 11(50.0) |  | 17(77.3) | 12(54.5) |  | 35.8 (20.3) | 34.4 (22.7) |  | N | N |  | 18 (81) | 14 (64) |
| Lookstein2020 | 155±91 | 151±94 |  | 107±62.9 | 110±68.8 |  | N | N |  | N | N |  | N | N |
| Maleux2018 | N | N |  | N | N |  | 2.1±1.2 | 2.2±1.2 |  | 30±24 | 30±38.4 |  | N | N |
| Moreno2019 | 46(65.7) | 55(70.5) |  | 50(71.4) | 60(76.9) |  | N | N |  | N | N |  | 41(58.5) | 32(41.5) |
| Roosen2017 | 7 | 7 |  | 6 | 4 |  | 2(2–3.75) | 3(1.75–4) |  | N | N |  | 9 | 10 |
| Swinne2019 | 12(17.6) | 6(10.0) |  | 38(55.9) | 39(65.0) |  | 2.2±2.2 | 1.9±1.3 |  | 3 (4.4) | 5 (8.3) |  | N | N |
| Trerotola2018 | 133 | 142 |  | 82 | 94 |  | N | N |  | 41±33 | 36±31 |  | N | N |
| Yin2021 | 66 | 70 |  | 27 | 29 |  | 19.1 ± 10.4 | 19.1 ± 9.7 |  | N | N |  | N | N |

N: not clear. Data presented as median (interquartile range) or n of patients (%).

**Table S2. Search strategy.**

| **Database** | **No** | **PICOS** | **Strategy** |
| --- | --- | --- | --- |
| PubMed | #1 | P | Renal Dialysis [MeSH] OR Dialysis OR Hemodialysis OR Haemodialysis OR ESRD OR Hemodialyses OR Peritoneal Dialysis |
|  | #2 | I | Drug Coated Balloon [MeSH] OR Drug-coated balloon OR DCB OR Paclitaxel-coated balloon OR drug-eluting balloon |
|  | #3 | C | Common Balloon [MeSH] OR POBA OR DCB OR Plain old balloon angioplasty OR CB |
|  | #4 | O | NA |
|  | #5 | S | (randomized controlled trial[Publication Type] OR randomized[TIAB] OR randomised[TIAB] OR placebo[TIAB]) NOT (Review[Publication Type]) NOT (meta-analysis[Publication Type]) NOT (Comment[Publication Type]) NOT (Letter[Publication Type]) |
|  | #6 = | NA | ((Renal Dialysis [MeSH] OR Dialysis OR Hemodialysis OR Haemodialysis OR ESRD OR Hemodialyses OR Peritoneal Dialysis) AND (Drug Coated Balloon [MeSH] OR Drug-coated balloon OR DCB OR Paclitaxel-coated balloon OR drug-eluting balloon) AND ((randomized controlled trial[Publication Type] OR randomized[TIAB] OR randomised[TIAB] OR placebo[TIAB]) NOT (Review[Publication Type]) NOT (meta-analysis[Publication Type]) NOT (Comment[Publication Type]) NOT (Letter[Publication Type])) |
| Embase | #7 | P | 'Dialysis'/exp OR 'hemodialysis':ab,ti OR 'ESRD':ab,ti OR ‘Haemodialysis’:ab,ti OR ‘Renal Dialysis’:ab,ti OR 'Renal Dialysis':ab,ti |
|  | #8 | I | 'Drug Coated Balloon'/exp OR 'Drug-coated balloon':ab,ti OR ' DCB ':ab,ti OR ' Paclitaxel-coated balloon':ab,ti OR ' drug-eluting balloon ':ab,ti |
|  | #9 | C | 'Common Balloon'/exp OR 'CB':ab,ti OR 'POBA':ab,ti OR 'Plain old balloon angioplasty ':ab,ti |
|  | #10 | O | NA |
|  | #11 | S | 'randomized controlled trial'/exp NOT review:it |
|  | #12 | NA | ('Dialysis'/exp OR 'hemodialysis': ab,ti OR 'ESRD': ab,ti OR ‘Haemodialysis’ :ab,ti OR ‘Renal Dialysis’:ab,ti OR 'Renal Dialysis':ab,ti) AND ('Drug Coated Balloon'/exp OR 'Drug-coated balloon':ab,ti OR ' DCB ':ab,ti OR ' Paclitaxel-coated balloon':ab,ti OR ' drug-eluting balloon ':ab,ti) AND ('Common Balloon'/exp OR 'CB':ab,ti OR 'POBA':ab,ti OR 'Plain old balloon angioplasty ':ab,ti) AND ('randomized controlled trial'/exp NOT review:it) |
| Web of Science | #13 | P | ((((ALL=(Renal Dialyses)) OR ALL=(Hemodialysis)) OR ALL=(Hemodialyses)) OR ALL=(ESRD)) OR ALL=(Dialysis) |
|  | #14 | I | ((((ALL=(Drug Coated Balloon)) OR ALL=(Drug-coated balloon)) OR ALL=(DCB)) OR ALL=(Paclitaxel-coated balloon)) OR ALL=(drug-eluting balloon) |
|  | #15 | C | (((ALL=(Common Balloon)) OR ALL=(CB)) OR ALL=(POBA)) OR ALL=(Plain old balloon angioplasty) |
|  | #16 | O | NA |
|  | #17 | S | ALL=(randomized controlled trial) |
|  | #18 | NA | #13 AND #14 AND #15 AND #17 |
| The Cochrane Library | #19 | P | MeSH descriptor: [Renal Dialysis] explode all trees |
|  | #20 |  | Hemodialysis or Hemodialyses or ESRD OR Dialysis |
|  | #21 |  | #19 or #20 |
|  | #22 | I | Drug Coated Balloon or Drug-coated balloon or DCB or Paclitaxel-coated balloon or drug-eluting balloon |
|  | #23 | C | Common Balloon or CB or POBA or Plain old balloon angioplasty |
|  | #24 | O | NA |
|  | #25 | S | NA |
|  | #26 | NA | #21 and #22 and #23 |

Abbreviation: NA not applicable

**Table S3. Inclusion/exclusion criteria of literature**

| **PICOS** | **Inclusion** | **Exclusion** |
| --- | --- | --- |
| P | Adults participants (≥18 y) were on dialysis for at least 1 year, irrespective of age, gender, and race. | Children, pregnant women, and patients with a history of kidney transplantation were excluded |
| I | Participants were on hemodialysis for at least 6 month, After the diagnosis of arteriovenous fistula stenosis, drug-coated balloon dilatation was performed | Not available |
| C | Participants were on hemodialysis for at least 6 month, After the diagnosis of arteriovenous fistula stenosis, common balloon dilatation was performed | Not available |
| O | One of the following outcomes must have been included: primary patency and all-cause mortality at 6 months or 12 months. | Not available |
| S | RCT irrespective of blinding or arm | 1) Articles without peer reviewed or unpublished  2) Studies that were repeatedly published or had qualitative outcomes  3) Quasi-experimental studies and observational studies |

| Quality assessment | | | | | | |  | Summary of Findings | | | |
| --- | --- | --- | --- | --- | --- | --- | --- | --- | --- | --- | --- |
| Outcomes | Study  design | Risk  of bias | Inconsistency | Indirectness | Imprecision | Publication  bias |  | No. of Patients | | Absolute  (95% CI) | Overall quality of evidence |
|  |  |  |  |  |  |  |  | Treatment | Control |  |  |
| Primary patency rate of the target lesion (6months) | RCT | no serious | no serious | no serious | no serious | no serious |  | 750 | 739 | 2.31 (1.69 to 3.15) | ⊕⊕⊕⊕  HIGH |
| Primary patency rate of the target lesion (12months) | RCT | no serious | no serious | no serious | no serious | no serious |  | 457 | 453 | 2.09 (1.50 to 2.91) | ⊕⊕⊕⊕  HIGH |
| All-cause mortality  (6months) | RCT | no serious | no serious | no serious | no serious | no serious |  | 382 | 375 | 0.85 (0.47 to 1.52) | ⊕⊕⊕⊕  HIGH |
| All-cause mortality  (12months) | RCT | no serious | no serious | no serious | no serious | no serious |  | 382 | 392 | 0.99 (0.60 to 1.64) | ⊕⊕⊕⊕  HIGH |

**Table S4. Summary of findings.**

GRADE Working Group grades of evidence
**High quality:** Further research is very unlikely to change our confidence in the estimate of effect.
**Moderate quality:** Further research is likely to have an important impact on our confidence in the estimate of effect and may change the estimate.
**Low quality:** Further research is very likely to have an important impact on our confidence in the estimate of effect and is likely to change the estimate.
**Very low quality:** We are very uncertain about the estimate.

Table S5. Inter-rater agreement for study selection and risk of bias.

|  | 95% confidence interval | Kappa | P | %agreement |
| --- | --- | --- | --- | --- |
| Study selection | 0.36-1.36 | 0.86 | 0.0007 | 93% |
| Random sequence generation (selection bias) | 0.40-1.89 | 0.84 | 0.0002 | 90% |
| Allocation concealment (selection bias) | 0.33-1.04 | 0.68 | <0.0001 | 80% |
| Blinding of participants and personnel (performance bias) | 0.44-1.15 | 0.79 | <0.0001 | 80% |
| Blinding of outcome assessment (detection bias) | 0.35-1.03 | 0.84 | <0.0001 | 80% |
| Incomplete outcome data (attrition bias) | 0.28-0.93 | 0.61 | 0.0002 | 90% |
